# *Moringa oleifera* leaf alleviates functional constipation via regulating the gut microbiota and the enteric nervous system in mice

**DOI:** 10.3389/fmicb.2023.1315402

**Published:** 2023-12-20

**Authors:** Xiaoyu Gao, Weiqian Yang, Sijin Li, Shuangfeng Liu, Weixing Yang, Shuang Song, Jun Sheng, Yan Zhao, Yang Tian

**Affiliations:** ^1^Yunnan Key Laboratory of Precision Nutrition and Personalized Food Manufacturing, Yunnan Agricultural University, Kunming, China; ^2^College of Food Science and Technology, Yunnan Agricultural University, Kunming, China; ^3^Engineering Research Center of Development and Utilization of Food and Drug Homologous Resources, Ministry of Education, Yunnan Agricultural University, Kunming, China; ^4^Department of Hotel Management, Chongqing Vocational Institute of Tourism, Chongqing, China; ^5^College of Pu’er Tea West Yunnan University of Applied Sciences, Puer, China; ^6^Division of Science and Technology, Yunnan Agricultural University, Kunming, China

**Keywords:** intestinal motility, gut microbiota, *Bacteroides*, *Butyricoccus*, *Ruminococcus*, *Desulfovibrio*

## Abstract

*Moringa oleifera* Lam. leaf is not only a new food resource in China, but also a traditional medicinal plant. It is commonly used in the folk to alleviate constipation, but its laxative mechanism is not fully understood. Hence we investigated it in loperamide-induced functional constipation (FC) mice. The results showed that MOAE significantly regulated not only gastrointestinal hormones and neurotransmitters in serum but also important gastrointestinal motility factors in the enteric nervous system (ENS)-interstitial cells of Cajal (ICCs)-smooth muscle cell (SMC) network. Meanwhile, MOAE attenuated intestinal inflammation, increased cecal short-chain fatty acid levels and colonic antimicrobial peptide expression, and improved the impaired intestinal barrier function in loperamide-induced FC mice. In addition, MOAE also increased fecal water content by inhibiting the mRNA expression of colonic aquaporins (*Aqp3* and *Aqp4*) in FC mice. Interestingly and importantly, MOAE affected the intestinal microbiota by inhibiting some key “constipation-causing” microbiota, such as Bacteroidaceae, Clostridiaceae, *Bacteroides*, and *Ruminococcus*, and promoting the growth of other important “constipation-curing” microbiota, such as *Butyricoccus*, *Tyzzerella*, and *Desulfovibrio*. These important taxa are significantly associated with a variety of indicators of constipation. These findings suggest that MOAE can promote defecation through its rich chemical composition to modulate the ENS-ICCs-SMCs network and the gut microecosystem.

## Graphical abstract

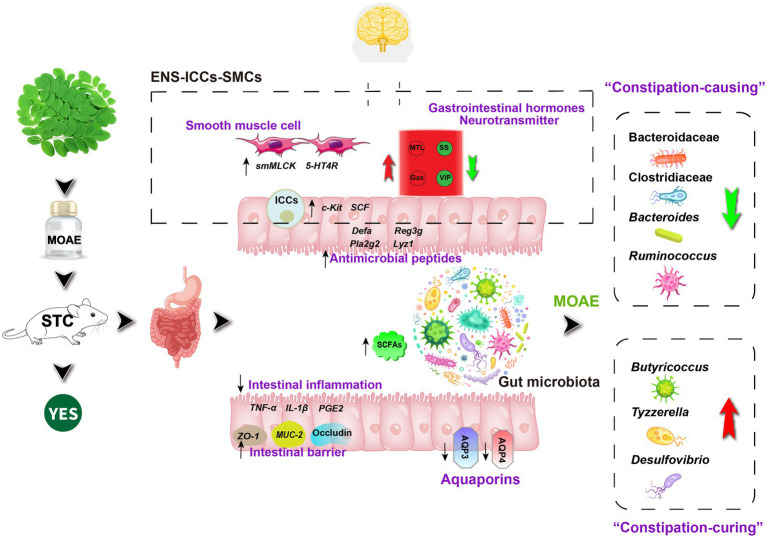

## Highlights

- MOAE can relieve the loperamide-induced FC by modulating ENS-ICCs-SMCs network.
- MOAE can restore the gut microbiota imbanlance induced by loperamide.
- Gut microbiota regulated by MOAE was correlated with constipation phenotype.

## Introduction

Functional constipation (FC) is a common gastrointestinal disorder in the population, and its prevalence has been shown to vary in cross-sectional surveys (Barberio et al., 2021). It can be divided into three subgroups based on different pathological mechanisms, including defecation disorder, slow transit constipation (STC), and normal transit constipation (Vriesman et al., 2020). Human defecation is a complex physiological movement process that involves multiple systems in the body; disorder in any link will lead to constipation. FC is multifactorial in its pathophysiology. General pathophysiology includes genetic factors, parental factors, psychological and behavioral factors, lifestyle factors, colonic motility factors, anorectal factors, and the microbiome (Vriesman et al., 2020).

The treatment of FC remains a difficult problem. The limitations of conventional drugs provide opportunities for the application of important traditional resources including both medicine and food. Native to western and sub-Himalayan regions, *Moringa oleifera* Lam.(MO) is known for its diverse medicinal and nutritional uses (Gao et al., 2017). As a traditional medicinal plant, it has been used in folk medicine to relieve constipation (Senthilkumar et al., 2018). However, MO leaves were also authenticated as new food resources. Based on pharmacodynamics and serum metabolomics, Li C. et al. found that *M. oleifera* leaves have long-lasting and mild laxative effects, increase the defecation volume and water content of feces, and may be a potential medicine for treating constipation (Li C. et al., 2022).

Accumulating evidence has highlighted the link between imbalances in the gut microbiota and constipation (Pan et al., 2022). The development and maturation of the enteric nervous system (ENS) has been shown to depend on bacterial colonization of the gut (Joly et al., 2021). The ENS is structurally similar to the central nervous system, as it independently regulates neurons for gut motility and brain-gut communication (Liu et al., 2009). Disruption of the microbiota-gut-brain axis signalling may lead to altered gut motility (Kennedy et al., 2012; Carabotti et al., 2015). Some important microbes can influence defecation behavior by regulating intestinal motility, such as *Lactobacillus rhamnosus*, *L Lactobacillus paracasei* and *Bacillus coagulans* (Chen et al., 2020; Wang G. et al., 2020; Zhou et al., 2022). LPS and metabolites of gut microbes, such as deoxycholic acid and Short-chain fatty acids (SCFAs), can also affect intestinal motility (Rebollar et al., 2002; Fukumoto et al., 2003; Yano et al., 2015).

Although intestinal microecology is closely related to constipation, there is no report on whether *M. oleifera* leaves affect defecation behavior by regulating intestinal microecology. Therefore, we evaluated the laxative effect of *M. oleifera* leaf aqueous extract (MOAE) in loperamide-induced slow transit constipation (STC) mice and systematically explored the laxative mechanism of MOAE from the aspects of neurotransmitters, gastrointestinal hormones, intestinal motility related factors, gut aquaporins, intestinal inflammation, gut barrier factors, intestinal antimicrobial peptides, the gut microbiota and their metabolites.

## Materials and methods

### Preparation and chemical composition of *Moringa oleifera* leaf aqueous extract

The dry MO leaf powder was provided by Yunnan Tianyou Technology Development Co., Ltd. Dry *M. oleifera* leaf powder (400 g) was accurately weighed, exposed to boiled water (95°C, RO water) for 1 min at a ratio of 1:9 (g/mL), and then filtered immediately. After filtration was completed, the filtrate was continuously centrifuged three times in a high-speed centrifuge (4,500 rpm/m for 5 min), and the supernatant was taken. The filter residue was poured into boiling water for 1 min and then filtered immediately. The above step was repeated twice. After three rounds of centrifugation, the supernatants were poured together and vacuum freeze-dried to obtain *M. oleifera* leaf aqueous extract (MOAE). The yield was 20.0%. The methods listed in Supplementary Table S1 were used to determine the main nutritional components and phytochemical composition of MOAE.

### Experimental design for animal study

Seventy-two male C57BL/6 J mice (5–6 weeks old, 20–22 g) were purchased from Liaoning Changsheng Biotechnology Co., Ltd. The mice were housed in a controlled environment (24 ± 1°C, 12-h daylight cycle, lights off at 20,00) with *ad libitum* access to food and water. Mice were divided into the following six groups (*n* = 12) after adaptive feeding for 7 days, including the control group (CON, saline solution), the loperamide model group (LOP, 4 mg/kg loperamide), the positive control group (POS, 4 mg/kg loperamide and 900 mg/kg Maren pill from Beijing Tongrentang Pharmaceutical Co., Ltd.), the low-dose MOAE group (LMO, received 4 mg/kg loperamide and 250 mg/kg MOAE), the middle-dose MOAE group (MMO, received 4 mg/kg loperamide and 500 mg/kg MOAE), and the high-dose MOAE group (HMO, received 4 mg/kg loperamide and 750 mg/kg MOAE). All groups received daily oral gavage with the corresponding test substance at 9:30 a.m. for 7 days. During the experiment, the mice had *ad libitum* access to water, the body weight and food intake were monitored once every 2 days, and the padding and drinking water were changed every 4 days. Animal Ethics Committee of Yunnan Agriculture University approved the protocols of animal experiments (No.: 202001025).

### Defecation test

On the evening of the sixth day, the mice were fasted overnight for 12 h. All groups were subjected to oral gavage with the corresponding test substance at 8:00 a.m. the next day. Sixty minutes later, all mice were administered approximately 0.4 mL ink, placed in empty, clean cages and watered *ad libitum*. The defection time of the first black stool (FBST), the fecal number (FN) and the fecal wet weight (FW) were observed and recorded over 6 h. The feces of each mouse were collected and dried in an oven at 80°C for 36 h, and the fecal water content (FWC) was calculated. All these defecation parameters were used to assess the laxative effect. After the defection test, all the mice were placed back into their original cages for the gastrointestinal transit test the next day.

### Gastrointestinal transit test and tissue collection

On the evening of the seventh day, the mice were fasted overnight for 12 h. All groups were subjected to oral gavage with the corresponding test substance at 8:00 a.m. the next day. Sixty minutes later, all mice were gavage administered approximately 0.4 mL ink. Twenty minutes later, the mice were sacrificed to collect whole blood and the small intestinal segments, and then the ink advance distance was measured to calculate the gastrointestinal transmission rate (GTR). Serum was prepared by incubating the collected blood at 37°C for 30 min and centrifuged at 4°C and 3,500 rpm for 10 min. The distal ileum and proximal colon of each mouse were precisely dissected simultaneously, and their contents were rinsed with cold PBS. They were then snap frozen in liquid nitrogen and stored at -80°C for further use.

### Enzyme-linked immunosorbent assay

The levels of motilin (MTL) and vasoactive intestinal peptide (VIP) in serum were examined using kits purchased from Sangon Biotech (Shanghai, China). The levels of gastrin (Gas) and somatostatin (SS) in serum were measured using mouse Enzyme-linked immunosorbent assay (ELISA) kits obtained from Cusabio Biotech Co., Ltd. (Wuhan, China).

### Histopathology investigation

10% neutral formaldehyde fixative were used to fixed the ileum and colon of mice. Tissues were sectioned at 5 mm thickness and embedded in paraffin. Hematoxylin and eosin (H&E) staining was performed on 5-μm paraffin sections. An Olympus CX43 microscope with CellSens Entry software was used to capture the H&E staining images.

### RNA preparation and quantitative PCR analysis of gene expression

Total RNA was extracted from frozen tissue of both the ileum and colon by using the TaKaRa MiniBEST Universal RNA Extraction Kit (TaKaRa, 9,767, Dalian), and then complementary DNA was obtained by using the PrimeScript™ RT Reagent Kit with gDNA Eraser (Perfect Real Time) (TaKaRa, RR047A, Dalian). Quantitative PCR analysis of gene expression was conducted using a LightCycler/LightCycler480 System (Roche Diagnostics) with TB Green™ Premix ExTaq™II (TaKaRa, RR820A, Dalian) in both the ileum and colon. The 2^-△△Ct^ method was used for data analysis. The primer sequences are presented in Supplementary Table S2.

### Metabolite extraction and GC–MS analysis of short-chain fatty acids

A 0.10 mL sample was added into 1.5 mL EP tubes, 0.05 mL of 50% H_2_SO_4_ and 0.2 mL of 2-methylvaleric acid (25 mg/L stock in methyl tertbutyl ether) were added as an internal standard; the samples were vortexed for 30 s, oscillated for 10 min, and then ultrasonicated for 10 min (incubated in ice water). After centrifuging for 15 min at 10,000 rpm and 4°C, the mixture was kept at −20°C for 30 min. For GC–MS analysis, the supernatant was then transferred to a fresh 2 mL glass vial. An Agilent 7890B gas chromatograph coupled to an Agilent 5977B mass spectrometer was used in the assay, and the specific parameters were in general agreement with a previous report (Wang Y. et al., 2020). The content of various short-chain fatty acids (SCFAs) in the samples was calculated by constructing standard curves.

### 16S rRNA gene sequencing and bioinformatics analysis of the gut microbiota

Metagenomic DNA from the cecal content was extracted using a QIAamp-DNA stool mini-kit (Qiagen, 51,604, Germany). DNA samples were then shipped on dry ice to Majorbio Biotechnology Co., Ltd. (Shanghai, China). The detailed methods for sequencing gut microbiota 16S rRNA genes and bioinformatic analysis were performed using our previously described methods (Gao et al., 2020; Gao X. et al., 2022; Hu et al., 2023).

### Statistical analysis

The data are presented as the means ± standard errors of the means (SEMs). The Student’s *t-*test (unpaired two-tailed) was performed to analyze two independent groups. Spearman’s *r* coefficients were used for bivariate correlations. The heatmaps were generated using HemI 1.0 software. Unless otherwise stated in the figure legends, the results were considered statistically significant when the value of *p* is less than 0.05.

## Results

### Phytochemical composition and nutritional components of MOAE

As shown in Supplementary Table S1, the macronutrients included water (6.92%), fat (1.75%), protein (20.20%), carbohydrate (54.33%), and crude polysaccharide (5.14%). MOAE was also rich in mineral elements and vitamin C (720 mg/kg). The ash and total acid contents were 16.80 and 0.40%, respectively. Supplementary Table S3 shows the most abundant chemical compound categories found in MOAE. Flavonoids, amino acids, nucleotides, lipids, and alkaloids were the main classifications. There were 23 monomes with relative abundances greater than 0.50% (Supplementary Table S4). The monomes with a abundance of more than 5% were L-phenylalanine (11.84%), isoquercitrin (6.38%), astragalin (8.73%), and vidarabine (5.33%).

### MOAE alleviated the symptoms of constipation induced by loperamide in mice

**Figure 1:**
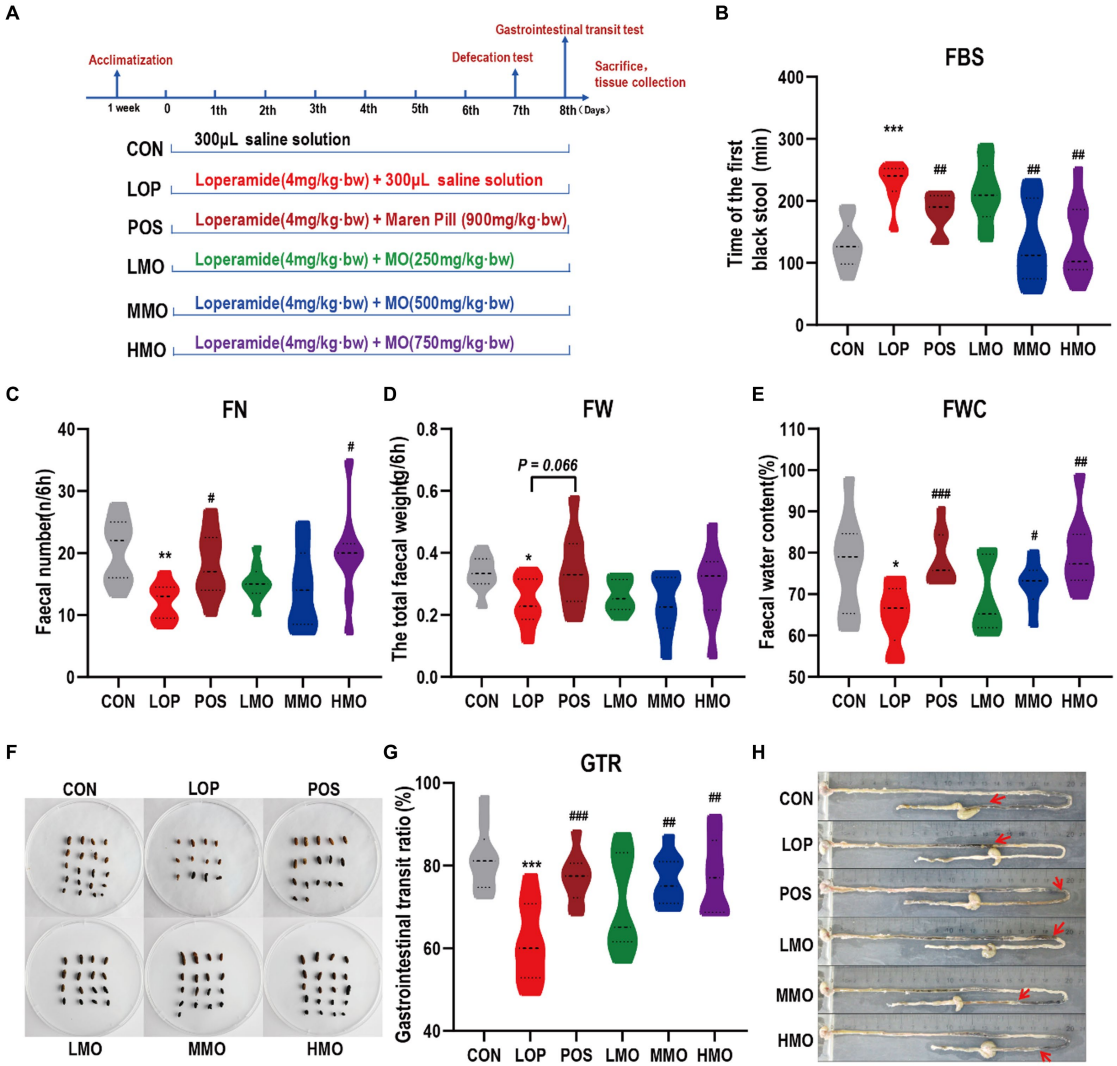
Effects of MOAE on the symptoms of FC in C57BL/6J mice. **(A)** Basic workflow and grouping; **(B)** The defecation time of the first black stool, FBST; **(C)** The fecal number, FN; **(D)** The fecal wet weight, FW; **(E)** The fecal water content, FWC; **(F)** The representative fecal morphology; **(G)** The gastrointestinal transit rate, GTR; **(H)** Representative pictures of ink advance. The data are presented as the mean ± SEM (*n* = 10–12). *, vs. CON group; #, vs. LOP group. **p < 0.01; ***p < 0.001. #*p* < 0.05; ##*p* < 0.01; ###*p* < 0.001.

To assess the laxative effect of MOAE, defecation tests and gastrointestinal transit tests were carried out (Figure 1A). In the defecation tests, we examined FBST, FN, FW and FWC within 6 h. Our results showed that the LOP group had a longer FBST than the CON group (*p* < 0.001), and the FBST was shorter in the MMO and HMO groups than in the LOP group (*p* < 0.01) (Figure 1B). Similarly, the FN and FWC were significantly higher in the HMO group than in the LOP group (*p* < 0.05) but there was not a significant increase in FW (Figures 1C–E). The fecal morphology in these groups, except the LOP group, was similar to that in the normal group; meanwhile, the feces appeared to have a larger bulk and higher surface moisture in the HMO group (Figure 1F).

In the gastrointestinal transit tests, loperamide effectively inhibited the movement of the small intestine (*p* < 0.001), and medium and high doses of MOAE significantly reversed the inhibitory effect of loperamide on intestinal motility (Figures 1G,H*, P* < 0.01). Notably, we also investigated the effect of MOAE on body weight (Supplementary Figure S1A), feeding behavior (Supplementary Figures S1B,C), and the organ index (Supplementary Figures S1D–H) of FC mice. MOAE treatment did not show any adverse effects on these parameters. These results suggested that MOAE could alleviate FC by promoting small intestinal peristalsis, shortening the defecation time, and increasing the water content of feces. The effect of MOAE was comparable to that of the positive drug.

### MOAE reversed loperamide-induced gastrointestinal hormone and neurotransmitter secretion abnormalities

**Figure 2:**
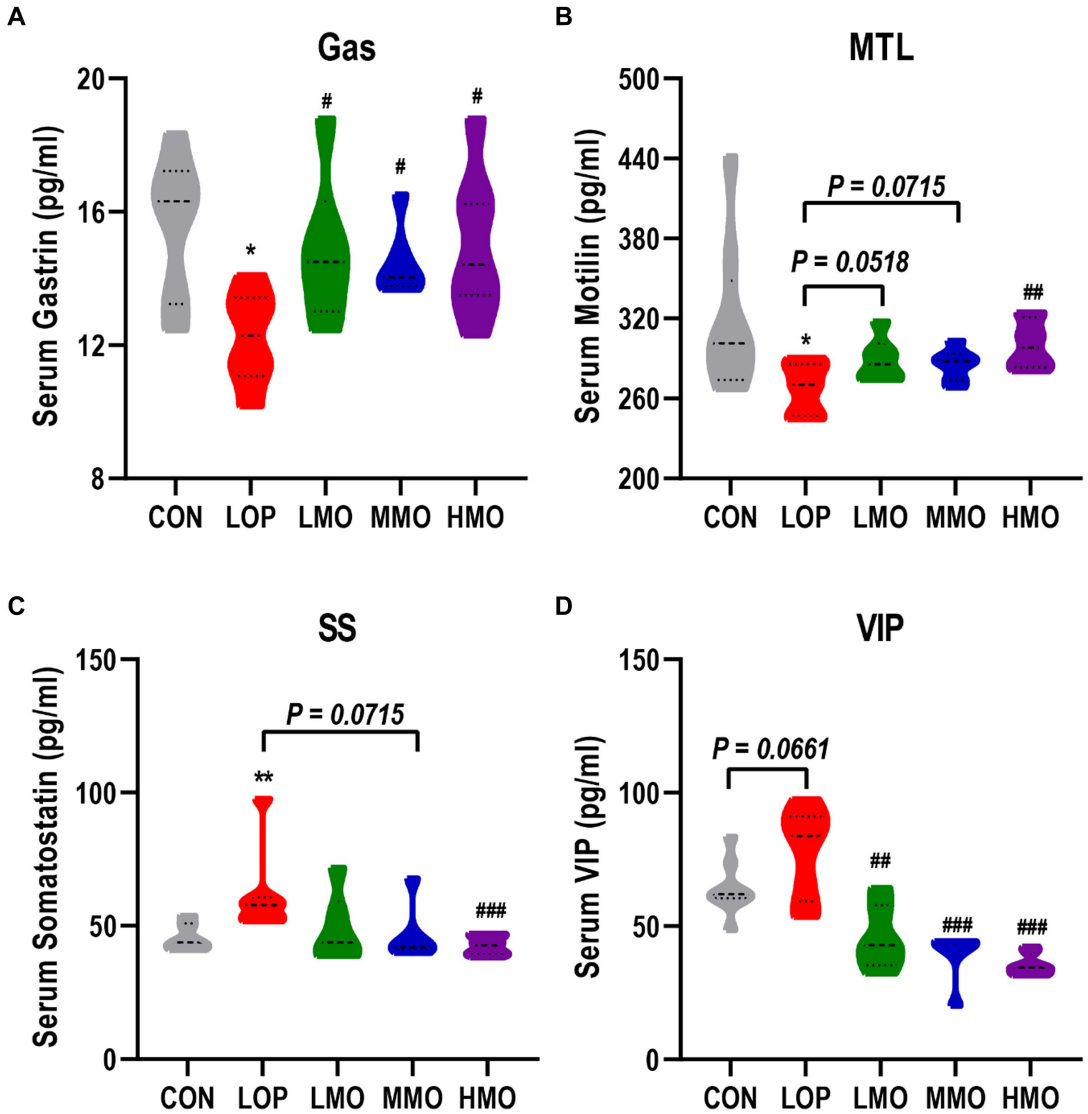
The effect of MOAE on the serum levels of neurotransmitters and gastrointestinal hormones. **(A)** Gastrin, **(B)** Motilin, **(C)** Somatostatin, and **(D)** vasoactive intestinal polypeptide levels in mice serum. The data are presented as the mean ± SEM (*n* = 8). *, vs. CON group; #, vs. LOP group. *p < 0.05; **p < 0.01. #*p* < 0.05; ##*p* < 0.01; ###*p* < 0.001. MOEA enhanced the expression of gastrointestinal motility-related factors.

Gastrin (Gas), motilin (MTL), Vasoactive intestinal polypeptide (VIP) and somatostatin (SS) are important neurotransmitters and gastrointestinal hormones. As important components of ENS, they play important roles in the regulation of gastrointestinal motility. Gas, MTL, SS, and VIP levels in serum were measured by ELISA. Loperamide significantly decreased the contents of the excitatory gastrointestinal hormones Gas and MTL (Figures 2A,B, *P* < 0.05), significantly increased the content of the inhibitory gastrointestinal hormone SS (Figure 2C, *P* < 0.01), and increased the content of the inhibitory neurotransmitter VIP (Figure 2D, *P* = 0.066). Different doses of MOAE reversed loperamide-induced gastrointestinal hormone and neurotransmitter disorders to varying degrees, and the HMO group showed the best regulation.

**Figure 3:**
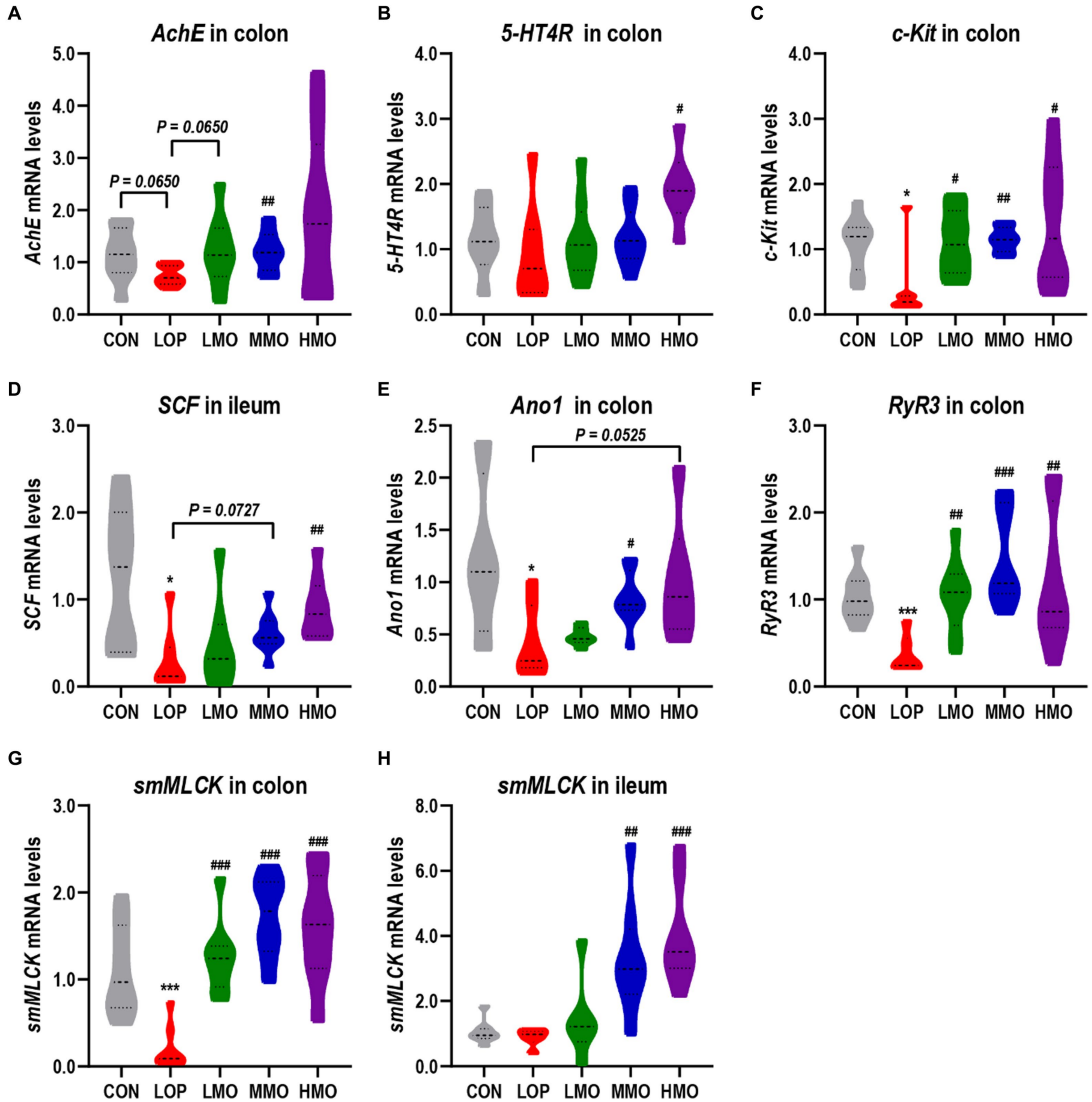
MOAE increased the mRNA expression of neurotransmitter receptors and gastrointestinal motility factors in mice. **(A–C)** The mRNA expression of *AchE*, *5-HT4R*, and *c-Kit* in the colon; **(D)** The mRNA expression of *SCF* in the ileum; **(E–G)** Colonic mRNA expression of *Ano1*, *RyR3*, and *smMLCK*; **(H)** The mRNA expression of *smMLCK* in the ileum. The data are presented as the mean ± SEM (*n* = 8). *, vs. CON group; #, vs. LOP group. *p < 0.05; ***p < 0.001. #*p* < 0.05; ##*p* < 0.01; ###*p* < 0.001.

5-Hydroxytryptamine (5-HT) and acetylcholine (Ach) are both exciting neurotransmitters that can stimulate bowel muscle contractions and gastrointestinal smooth muscle peristalsis. In our experiments, the mRNA expression of acetylcholinesterase (*AchE*) and 5-hydroxytryptamine receptor 4 (*5-HT4R*) in the colon could be significantly upregulated by treatment with a medium dose of MOEA and a high dose of MOEA, respectively (Figures 3A,B).

Interstitial cells of Cajal (ICCs) are the pacemaker cells of smooth muscle. The stem cell factor (SCF) and stem cell factor receptor (c-Kit) are important malers of ICCs. Our results showed that the mRNA expression of *c-Kit* in the colon and *SCF* in the ileum were significantly inhibited by loperamide, while high-dose MOAE treatment significantly upregulated their expression (Figures 3C,D).

The opening of the ryanodine receptor 3 (RyR3) can cause smooth muscle contraction through the activation of myosin light chain kinase (MLCK); in addition, anoctamin 1 (Ano1), a calcium-activated chloride channel, is also present in ICCs and plays an important role in intestinal motility (Gao X. et al., 2022). Similarly, loperamide significantly reduced the expression of *Ano1*, *RyR3* and smooth muscle myosin light chain kinase (*smMLCK*), whereas MOAE reversed their expression to different extent (Figures 3E–H), even to levels exceeding that of the CON group (Figures 3F–H). In all, MOAE could promote defecation by enhancing the expression of key factors associated with the motility of gastrointestinal tract.

### MOAE alleviated intestinal inflammation and damaged gut barrier function induced by loperamide

**Figure 4:**
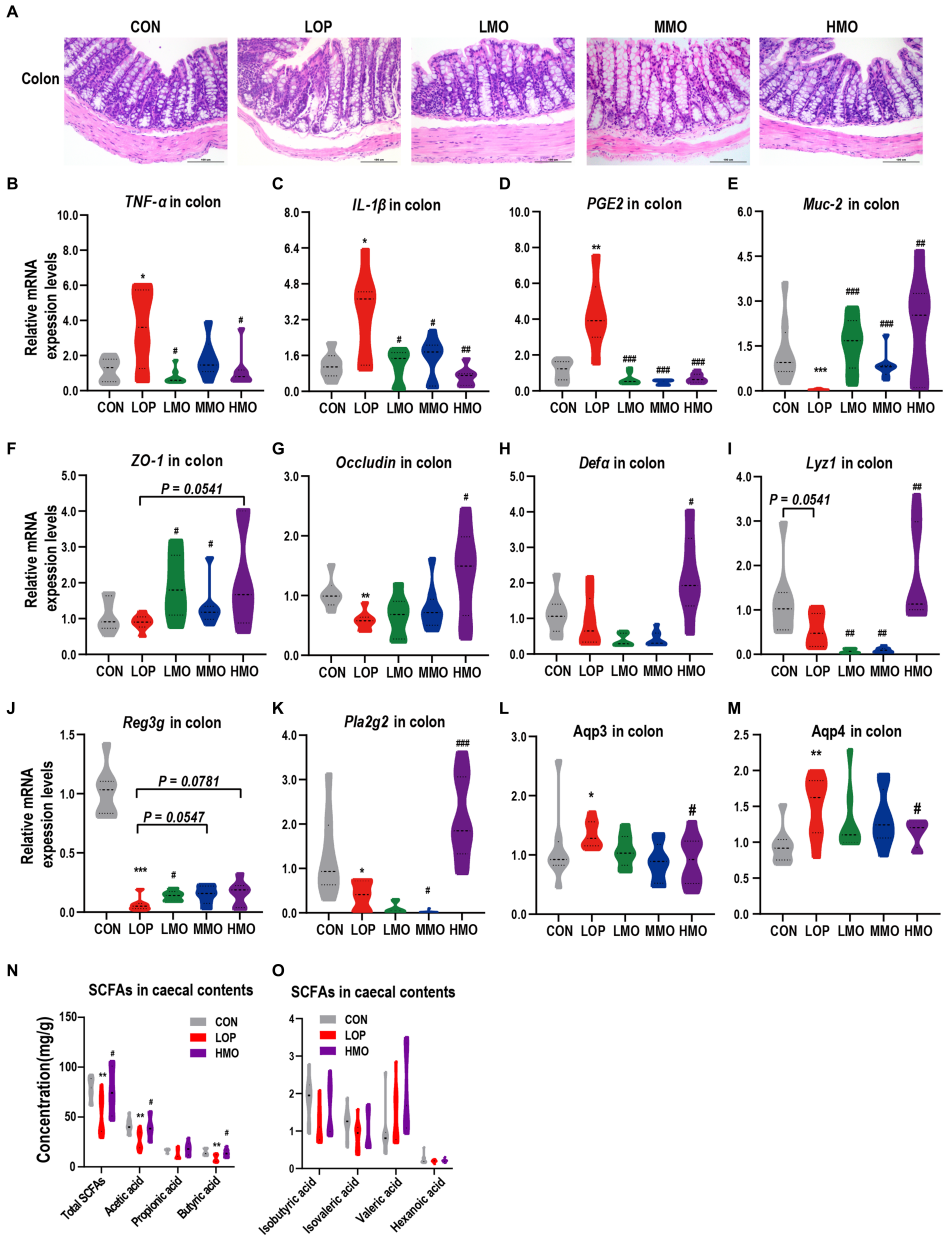
Effects of MOAE on intestinal inflammation, barrier, aquaporins and SCFAs in loperamide-induced FC mice. **(A)** Representative photographs of H&E staining of proximal colon sections. **(B–D)** Expression of *TNF-α*, *IL-1β*, and *PGE2* in the colon. **(E–G)** Expression of *Muc2*, *ZO-1*, and *Occludin* in the colon. **(H–K)** Expression of *Defa*, *Lyz1*, *Reg3g*, and *Pla2g2* in the colon. **(L,M)** Expression of the aquaporins (*Aqp3* and *Aqp4*) in the colon. **(N)** SCFAs in cecal contents, including total SCFAs, acetic acid, propionic acid, and butyric acid. **(O)** SCFAs in ceacal contents, including isobutyric acid, isovaleric acid, valeric acid, and hexanoic acid. The data are presented as the mean ± SEM (*n* = 6 or 8). *, vs. CON group; #, vs. LOP group. *p < 0.05; ***p < 0.001. #*p* < 0.05; ##*p* < 0.01; ###*p* < 0.001.

The process of functional constipation is often accompanied by intestinal inflammation and impaired intestinal barrier function (Vriesman et al., 2020). Colonic H&E staining and pro-inflammatory factor mRNA assay showed that loperamide induced inflammatory cell infiltration and high expression of pro-inflammatory factors (Figure 4A), while MOAE treatment inhibited the expression of proinflammatory cytokines (Figures 4B–D and Supplementary Figures S2K–L). Meanwhile, MOAE also restored the downregulation of several intestinal barrier function factors by loperamide, including *Muc2*, *ZO-1*, and *Occludin* (Figures 4E–G).

Antimicrobial peptides (AMPs) in the gut are important for defense against pathogens and for maintaining the homeostasis between the microbiota and the host (Gao et al., 2020). Loperamide reduced all the colonic mRNA expression of alpha-defensins (*Defa*), lysozyme C (*Lyz1*), regenerating islet-derived 3-gamma (*Reg3g*) and phospholipase A2 group II (*Pla2g2*) to varying degrees (Figures 4H–K); interestingly, high doses of MOAE significantly up-regulated these AMPs (Figures 4H–K), except for *Reg3g*, for which the change was not statistically significant (Figure 4J, *p* = 0.0781). Notably, LOP and MOAE had similar effects on the mouse ileum, but the overall effects were not as significant as their effects on the colon (Supplementary Figures S2A–J). This may be due to the fact that different intestinal segments have different histological structures, intestinal microecological environments and levels of MOAE utilization. In summary, MOAE alleviated intestinal inflammation and damaged gut barrier function in loperamide induced FC mice.

### Effects of MOAE on colonic aquaporins and cecal SCFAs in loperamide-induced FC mice

Given the ability of MOAE to increase fecal water content in loperamide-induced FC mice (Figure 1E), we examined the mRNA expression levels of two important aquaporins, *Aqp3* and *Aqp4*, in colonic tissue. Loperamide significantly up-regulated *Aqp3* and *Aqp4*, while a high dose of MOAE treatment reversed these changes, and the expression levels of *Aqp3* and *Aqp4* in the HMO group were close to those in the CON group (Figures 4L,M). At the same time, high-dose MOAE treatment also significantly reversed the decrease in the levels of total SCFAs, acetic acid, propionic acid, and butyric acid in the cecal content caused by LOP (Figure 4N). The effects of LOP and MOAE on several other SCFAs were not significant (Figure 4O). These results suggest that MOAE might improve the intestinal environment by regulating water balance and increasing the content of SCFAs.

### MOAE ameliorated loperamide-induced gut microbiota disorder in FC mice

**Figure 5:**
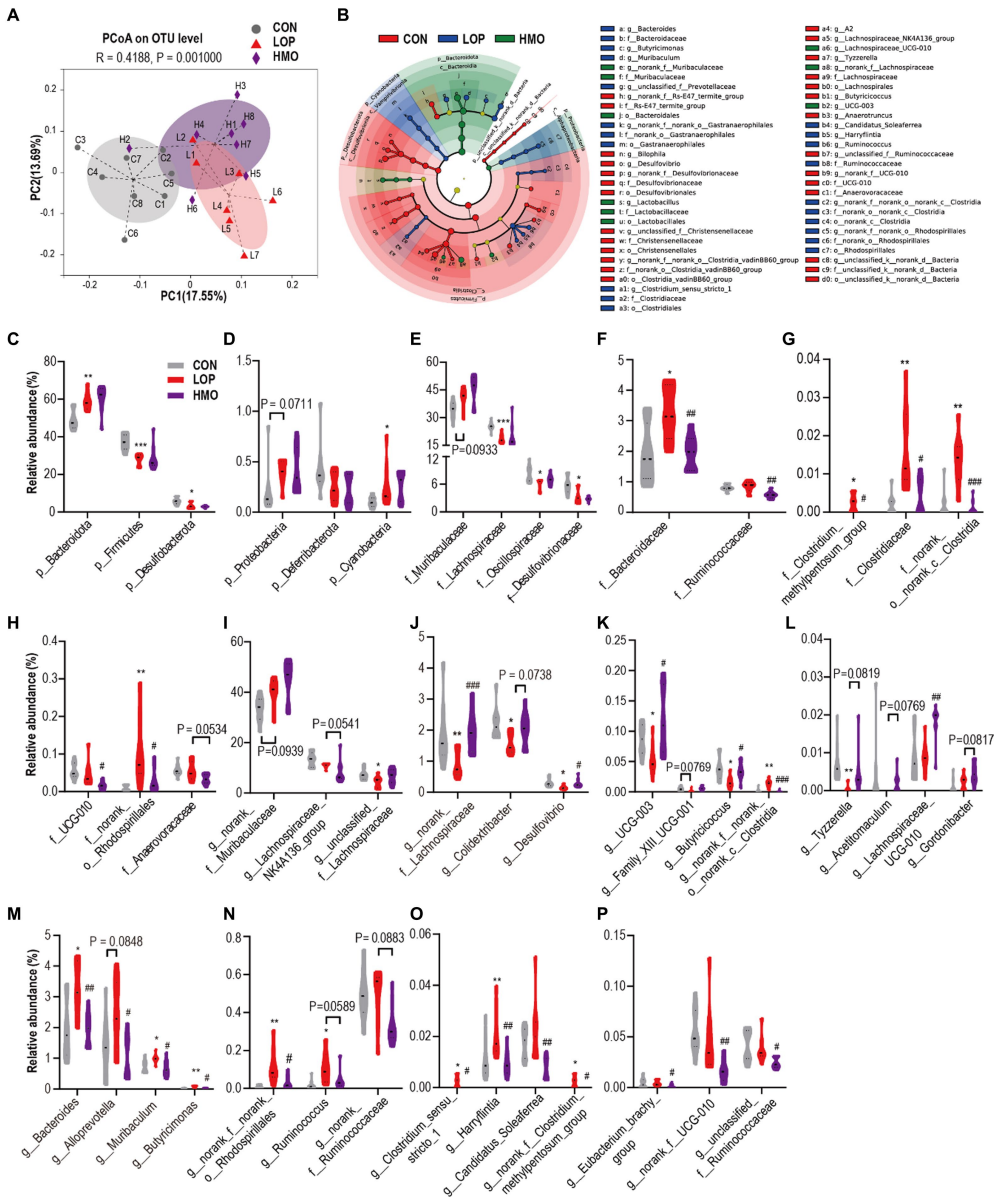
MOAE restored the gut microbial community structural and composition shift in FC mice **(A)** PCoA plot (Bray–Curtis dissimilarity). **(B)** LEfSe analyses (phylum to genera, LDA score > 2.0). **(C–P)** The relative abundance of gut microbiota at the phylum **(C,D)**, family **(E–H),** and genus levels **(I–P)**. The data are presented as the mean ± SEM, *n* = 7–8. * vs. NCD group, ^#^ vs. LOP group. ^*^p ≤ 0.05, ^**^p ≤ 0.01, ^***^p ≤ 0.001; ^#^*p* ≤ 0.05, ^##^*p* ≤ 0.01, ^###^*p* ≤ 0.001.

To assess the impact of loperamide and MOAE on the cecal microbiota, 16S rRNA gene sequencing was performed. We obtained 1,366,660 sequences from 23 samples in the three groups, and each sample contained more than 41,924 valid sequences, and identified 700 OTUs, 135 genera, 58 families, and 11 phyla. The Sobs index rarefaction curve of each sample indicated that the sequencing result was reliable (Supplementary Figure S3A). Although MOAE had a limited effect on α diversity (Supplementary Figures S3B–F), MOAE altered β diversity (Figure 5A). Meanwhile, MOAE changed the relative abundance of many cecal microbial taxa (Supplementary Figures S4A–C).

The dominant microbiota in each group was determined by linear discriminant analysis effect size (LEfSe) analyses (Figure 5B and Supplementary Figures S5A–D). It shows 69 different taxa in in the three groups (Supplementary Figures S5A–D). Our focus was on taxa that were significantly affected by loperamide or MOAE, particularly those with MOAE reversal (Supplementary Figures S5A–D).

At the phylum level, Bacteroidota, Firmicutes, Desulfobacterota and Cyanobacteria were significantly changed by loperamide treatment; however, MOAE had less impact on them (Figures 5C,D). At the family level, loperamide significantly altered the abundance of some dominant taxa (>1%, Figures 5E,F), including Lachnospiraceae, Oscillospiraceae, Desulfovibrionaceae, and Bacteroidaceae (*p* < 0.05); however, MOAE only significantly reversed the changes in the abundance of Bacteroidaceae. MOAE also significantly suppressed the increase in abundance of some relatively less abundant taxa induced by loperamide, such as Clostridium_methylpentosum_group, Clostridiaceae, norank_o_norank_C_Clostridia and norank_o_Rhodospirillales. Although the effects of loperamide on Ruminococcaceae and UCG-010 were small, MOAE treatment significantly reduced their abundance (Figures 5F,H).

At the genus level, *norank_f_Muribaculaceae* was the most abundant; however, neither loperamide nor MOAE had significant effects on this genera (Figure 5I). Loperamide reduced the relative abundance of two important taxa of Lachnospiraceae, *Lachnospiraceae_NK4A136_group* and *unclassified_f_Lachnospiraceae*. However, MOAE treatment restored the relative abundance of *unclassified_f_Lachnospiraceae* to control levels to some extent (Figure 5I). Compared with CON, loperamide also significantly reduced the abundance of *norank_f_Lachnospiraceae*, *Colidextribacter*, *Desulfovibrio*, *UCG-003*, *Family_XIII_UCG-001*, *Butyricicoccus*, *norank_f_norank_o_norank_c_Clostridia*, and *Tyzzerella* (Figures 5J–L), while MOAE significantly or nearly significantly increased the relative abundance of these taxa. Notably, MOAE also significantly increased the relative abundance of *Lachnospiraceae_UCG-010* in the caecum of FC mice, although loperamide had little effect on it (Figure 5L).

Loperamide significantly (or nearly significantly) increased the four genera of Bacteroidota (Figure 5M), *Bacteroides*, *Alloprevotella*, *Muribaculum*, and *Butyricimonas,* while MOAE significantly reduced the abundance of them. Loperamide also significantly increased the relative abundance of *norank_f_norank_o_ Rhodospirillales* (a genus of Proteobacteria), while MOAE treatment significantly decreased its relative abundance in the caecum of FC mice (Figure 5N). Meanwhile, loperamide increased *Ruminococcus*, *Clostridium_sensu_stricto_1*, *Harryflintia*, *Candidatus_Soleaferrea*, and *norank_f__Clostridium_methylpentosum_group* (genera of Firmicutes), while MOAE significantly (or nearly significantly) decreased their abundance (Figures 5N,O). MOAE also significantly reduced the relative abundance of *Eubacterium_brachy_group*, *norank_f_UCG-010*, and *unclassified_f_Ruminococcaceae* in the caecum of FC mice, although loperamide had little effect on these bacteria (Figure 5P).

### Correlations between the specific gut bacterial taxa and key host parameters

**Figure 6:**
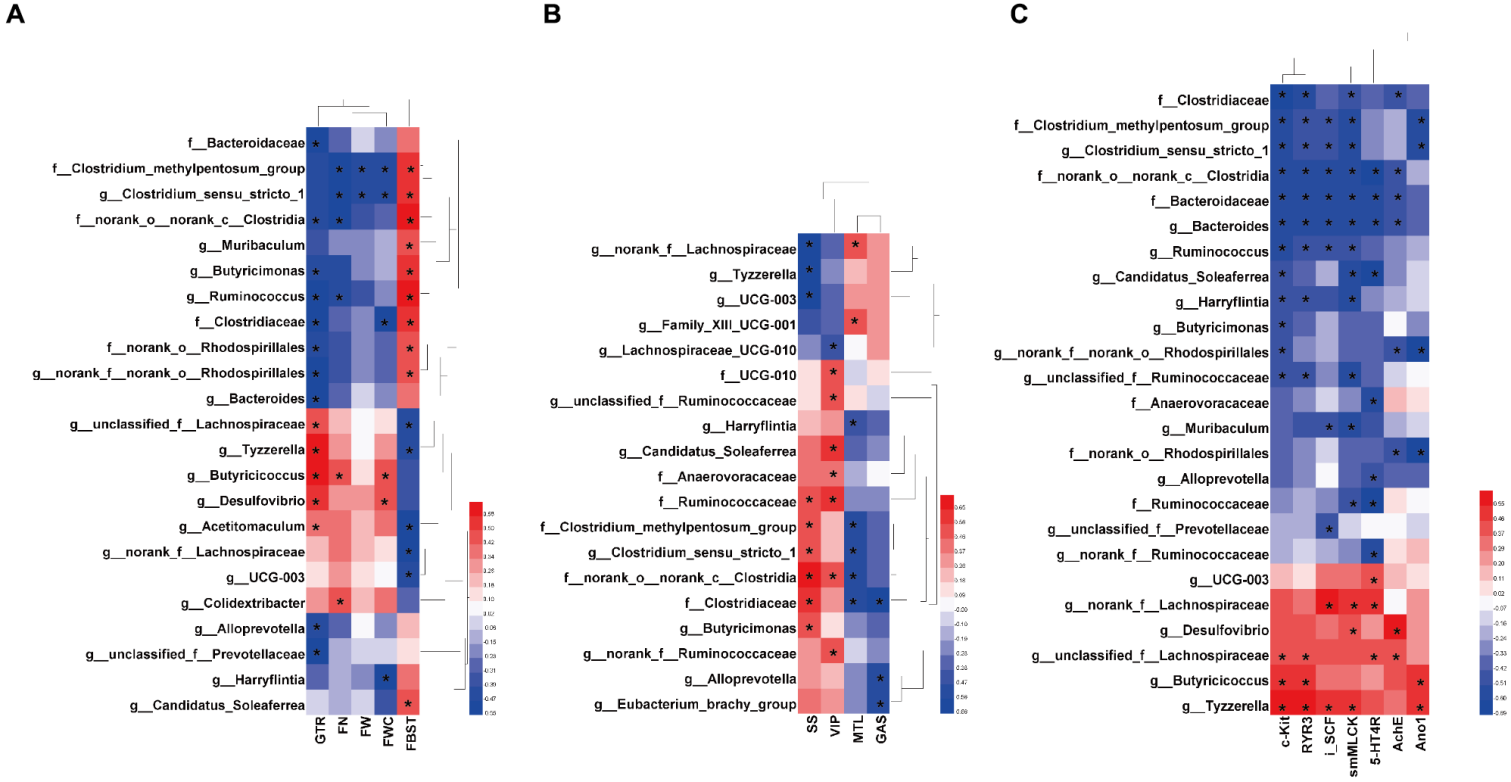
Heatmaps showing correlations between the specific gut bacterial taxa and key host parameters. **(A)** Correlations between the specific taxa and laxative phenotypic indicators, including FBST, FN, FW, FWC and GTR. **(B)** Correlations between gut bacteria and gastrointestinal hormones and neurotransmitters in serum, including GAS, MTL, SS, and VIP. **(C)** Correlations between the specific taxa and neurotransmitter receptors and intestinal motility related factors, including *AchE*, *5-HT4R*, *c-Kit*,*RyR3*, *smMLCK*, and *Ano1* in the colon, and *SCF* in the ileum (*iSCF*). The color at each point of intersection indicates the value of the *r* coefficient; The *Bonferroni* and *Hochberg* procedures were used to adjust *p-*values for multiple testing. * Indicates that there is a significant correlation between these two parameters (*p* < 0.05).

Based on the significant relief of constipation symptoms, gastrointestinal motility and the intestinal microecosystem in FC mice by MOAE, we established correlations between specific gut bacteria taxa (at the family and genus levels) and core host parameters (Figures 6A–C).

There are clear and strong correlations between specific taxa and laxative phenotypic indicators (Figure 6A). At the family level, Bacteroidaceae, Clostridiaceae, Clostridium_methylpentosum_group, norank_o_norank_c_Clostridia, and norank_o_Rhodospirillales were positively correlated with FBST or negatively correlated with GTR (*p* < 0.05). At the genus level, *Clostridium_sensu_stricto_1*, *Muribaculum*, *Butyricimonas*, *Ruminococcus*, *Bacteroides*, *Alloprevotella*, *unclassified_f_Prevotellaceae*, and *Candidatus_Soleaferrea* also showed similar correlations (*p* < 0.05). This suggests that these taxa might promote in the development of loperamide-induced constipation. Interestingly, MOAE reduced their relative abundance in the cecal microbiome of FC mice (Figures 5F–H,J,M–O). On the other hand, some taxa at the genus level showed opposite correlations, including *unclassified_f_Lachnospiraceae*, *Tyzzerella*, *Butyricicoccus*, *Desulfovibrio*, *Acetitomaculum*, *norank_f__Lachnospiraceae*, and *UCG-003.* Again, this suggests that these taxa might be potentially beneficial bacteria. Importantly, MOAE treatment increased their relative abundances (Figures 5I–L,O). Of course, other laxative phenotypes such as FN, FW and FWC were also strongly associated with these specific taxa. All of these numerous correlations support the important role of the gut microbiota in the relief of constipation by MOAE.

Correlations between the specific taxa and intestinal motility related factors, intestinal inflammation, gut barrier, and cecal SCFAs were also systematically analyzed. We found that Ruminococcaceae, Clostridium_methylpentosum_group, *Clostridium_sensu_stricto_1*, norank_o__norank_c_Clostridia, and Clostridiaceae were significantly correlated with more than two serum gut hormones (or neurotransmitters), and they were all positively correlated with SS and negatively correlated with MTL, while *norank_f_Lachnospiraceae*, *Tyzzerella* and *UCG-003* showed the opposite correlations (Figure 6B). Meanwhile, serum VIP and Gas also showed strong correlations with a variety of taxa (Figure 6B). Figure 6C clearly shows the correlations between 25 taxa and the mRNA expression of seven gastrointestinal motility-related factors in the colon. Clostridiaceae, Clostridium_methylpentosum_group, *Clostridium_sensu_stricto_1*, norank_o__norank_c__Clostridia, Bacteroidaceae, *Bacteroides*, and *Ruminococcus* were negatively and significantly correlated with more than four related factors. *Norank_f__Lachnospiraceae*, *Desulfovibrio*, *unclassified_f__Lachnospiraceae*, *Butyricicoccus*, and *Tyzzerella* were negatively and significantly correlated with more than two related factors. Colonic *c-kit* and *smMLCK* show more correlations with gut microbes. All these results supported the notion that MOAE could affect gastrointestinal motility by regulating these microbial groups.

Correlations between the specific taxa and intestinal inflammation are shown in Supplementary Figure S6A. Bacteroidaceae, Clostridium_methylpentosum_group, norank_o__norank_c__Clostridia, norank_o__Rhodospirillales, *Clostridium_sensu_stricto_1, Muribaculum*, *Bacteroides*, *norank_f__norank_o__Rhodospirillales* and *Ruminococcus* were all positively correlated with *TNF-α*, *IL-1β* and *PGE2* in the colon, while *Desulfovibrio*, *Acetitomaculum*, *UCG-003*, and *Lachnospiraceae_UCG-010* were negatively correlated with these proinflammatory factors.

The correlations between 18 taxa and three important gut barrier-related factors are shown in Supplementary Figure S6B. Among them, 13 taxa showed significant and negative correlations with *Occludin* or *Muc2*, including 5 families and 8 genera, which were norank_o__norank_c_Clostridia, Clostridium_methylpentosum_group, UCG-010, Bacteroidaceae, Clostridiaceae, *Butyricimonas*, *unclassified_f_Ruminococcaceae*, *Clostridium_sensu_stricto_1*, *Muribaculum*, *Alloprevotella*, *norank_f__norank_o_Rhodospirillales*, *Ruminococcus*, and *Bacteroides*. The other 5 taxa showed the opposite correlations and included *unclassified_f__Lachnospiraceae*, *Butyricicoccus*, *Tyzzerella*, *norank_f__Lachnospiraceae*, and *Desulfovibrio*. It is worth emphasizing that *Tyzzerella* showed the strongest correlation with gut barrier factors and was the only taxon that had a significant positive correlation with *ZO-1*.

Twenty-eight specific taxa also showed significant correlations with the expression of intestinal AMP genes (Supplementary Figure S6C). *Reg3g* shows the closest correlation with these microbial groups. Ruminococcaceae, norank_o__norank_c__Clostridia, Bacteroidaceae, *Candidatus_Soleaferrea*, *norank_f__Ruminococcaceae*, *Harryflintia*, *Muribaculum*, *Bacteroides*, *Alloprevotella*, and *unclassified_f__Ruminococcaceae* showed significant negative correlations with *Reg3g* in the ileum, while norank_o__Rhodospirillales, Clostridium_methylpentosum_group, Clostridiaceae, *norank_f__norank_o__Rhodospirillales*, *Ruminococcus*, *Clostridium_sensu_stricto_1*, and *unclassified_f__Prevotellaceae* showed significant negative correlations with *Reg3g* in the colon. Notably, *Tyzzerella*, *Butyricicoccus*, *Desulfovibrio*, *UCG-003*, and *Family_XIII_UCG-001* showed significant negative correlations with *Reg3g* in the colon or ileum.

In addition, we found significant correlations between SCFAs and some microbial groups (Supplementary Figure S6D). *Desulfovibrio* showed significant positive correlations with T-SCFAs, acetic acid and butyric acid, while *unclassified_f_Lachnospiraceae* showed significant positive correlations with only butyric acid. *Muribaculum*, *norank_f_norank_o_norank_c_Clostridia*, *Butyricimonas*, *norank_f_norank_o_Rhodospirillales*, *Ruminococcus*, *Harryflintia*, and norank_o_norank_c_Clostridia were all negatively correlated with T-SCFAs, acetic acid and butyric acid. Taken together, the preventive effect of MOAE in loperamide-induced FC mice can be explained in part by this complex but clear network that exists between specific gut bacterial taxa and key host parameters.

## Discussion

Functional constipation (FC) is a common, highly prevalent gastrointestinal disorder that significantly reduces patients’ quality of life. Humans have searched for many natural products from edible and medicinal plants that are effective in preventing and treating FC. Modern scientific research has confirmed the laxative effect of *M. oleifera* leaves (Li R. et al., 2022; Li C. et al., 2022; Li L. et al., 2022), but the mechanism of the laxative effect of *M. oleifera* leaves is not yet systematically understood. MOAE was found to be low in fat and high in protein and carbohydrates and contained a certain amount of polysaccharides (Supplementary Table S1). Polysaccharides from many plants have been reported to have significant laxative effects, such as *Durio zibethinus* rind, *Chrysanthemum morifolium*, and *Spirulina platensis* (Ma et al., 2019; Jiang et al., 2020; Wang et al., 2021).

All of these plant polysaccharides were capable of modulating the host intestinal microbiota while reducing constipation symptoms. *M. oleifera* leaf polysaccharides have various biological activities, such as the prevention of colitis and obesity, the enhancement of immunity, and the regulation of gut microbiota (Li et al., 2020; Li L. et al., 2022; Tian et al., 2022), which suggests that they may also have laxative effects, but further studies are needed to confirm this.

MOAE is also rich in vitamin C and mineral elements, including sodium, potassium, magnesium, calcium, phosphorus, iron, and zinc (Supplementary Table S1). Vitamin C has abundant biological activities, but its laxative properties have not been reported. Through a systematic literature review, we found that the presence of sodium, potassium, calcium, magnesium and phosphorus in nature in various forms have different degrees of relief for constipation, including calcium sennosides, magnesium oxide, magnesium sulfate, sodium hyaluronate, and potassium binder (Dupont et al., 2019; Ali et al., 2021; Mori et al., 2021; Imamura and Kinugawa, 2022; Vacca et al., 2022). In particular, dietary magnesium and phosphorus are closely related to the occurrence of constipation (Zhang et al., 2021; Zhao et al., 2023). Therefore, whether *M. oleifera* leaves exert laxative effects through their rich mineral elements is an interesting scientific question worthy of further investigation.

Flavonoids and amino acids are the two main classifications of the phytochemical compounds in MOAE (Supplementary Table S3). Flavonoids in *Allium mongolicum* Regel, *Amomum tsaoko* and Maojian green tea can relieve constipation (Chen et al., 2019; Hu et al., 2023; Wu et al., 2023). It is unknown whether the flavonoids in *M. oleifera* leaves have laxative effects. Isoquercitrin, astragalin, vitexin and rutin are the four flavonoids with relatively high abundance in MOAE. These four flavonoids have biological activities such as antioxidant, anti-inflammatory and gut microbiota regulation (Valentová et al., 2014; Riaz et al., 2018; Peng et al., 2021; Muvhulawa et al., 2022), but there is no report on their laxative activity thus far.

Phenylalanine (L-Phe) and L-arginine (L-Arg) are the two main amino acids in MOAE, with L-Phe being the monomeric compound with the highest relative abundance of 11.84% (Supplementary Table S4). Recent findings suggest that L-Phe can improve constipation in rats by remodeling the structure of the intestinal microbial community and altering metabolite levels (Yang et al., 2022). In addition, it has been reported that L-Phe can improve intestinal barrier health and the immune status of young grass carp, which is associated with regulating the gene expression of cytokines, tight junction proteins, and antioxidant enzymes (Feng et al., 2015). L-Phe is able to stimulate the secretion of gastric and humoral proteins in pigs through calcium-sensing receptors (Xian et al., 2018), and inhibit the activity of acetylcholinesterase in the brains and diaphragms of rats (Tsakiris et al., 1998; Tsakiris, 2001). These previous results support the idea that L-Phe is one of the important laxative compounds of MOAE.

L-Arg can be converted to NO *in vivo*, and there is also strong evidence that L-Arg supplementation can promote gastrointestinal motility and relieve constipation. For example, diarrhoea was found after oral administration of large amounts of L-Arg in clinical studies (Grimble, 2007), animal experimental studies also showed that intraperitoneal injection of L-Arg could reverse the constipation caused by morphine (Calignano et al., 1991), and long-term administration of L-Arg could also reverse the decline in gastrointestinal motility caused by morphine in mice (Karan et al., 2000). In addition, it has also been shown that dietary L-Arg can promote Aquaporin-3 expression and water transport in porcine trophoblast cells (Zhu et al., 2022) and attenuate colonic barrier damage, oxidative stress, and inflammation in intrauterine growth-retarded lactating lambs by modulating the gut microbiota (Zhang et al., 2022). In summary, MOAE may relieve constipation through a variety of components and pathways, only some of which we have discussed here, and more in-depth studies may be needed in the future.

Gastrointestinal hormones, neurotransmitters and gastrointestinal motility factors play an important role in the maintenance of gastrointestinal motility. They are often abnormally expressed in patients with constipation. Previous research results showed that MOAE could increase serum substance P and colonic GAS levels and decrease serum growth inhibitory hormone and colonic VIP levels in rats (Li et al., 2019). We also found that high doses of MOAE reversed loperamide-induced disturbances in gut hormones and neurotransmitters, including GAS, MTL, SS, and VIP, in serum, as well as the mRNA expression of *AchE* and *5-HT4R* in colonic tissues. These results fully confirmed that MOAE could promote the secretion of excitatory gastrointestinal hormones and neurotransmitters and reduce the secretion of inhibitory gastrointestinal hormones and neurotransmitters to promote gastrointestinal motility.

This study also examined the changes of important factors within the ENS-ICCs-SMCs network, which is primarily organized by ICCs (Gao X. et al., 2022). Acting as a kind of pacemaker cell, ICCs are facilitators of gastrointestinal electrical activity and regulators of neurotransmitter transmission (Zhu et al., 2021). The results of this study showed that high-dose MOAE treatment significantly upregulated *c-Kit* in the colon and *SCF* in the ileum (Figure 3). We also found that MOAE could restore loperidine-induced downregulation of *Ano1*, *RyR3* (a receptor in calcium channels) and *smMLCK* to varying degrees. These findings suggest that MOAE may alleviate FC by modulating the ENS-ICCs-SMCs network.

Patients with constipation often have impaired intestinal barrier function and varying degrees of intestinal inflammation. Previous studies have shown that a reduction in constipation symptoms is accompanied by the reduction of inflammation (Liu et al., 2020; Gao H. et al., 2022; Shatri et al., 2023). *M. oleifera* leaf aqueous extract was able to alleviate high-fat diet-induced intestinal inflammation (Mohamed Husien et al., 2022). *M. oleifera* polysaccharide aided intestinal barrier maintenance and reduced symptoms of experimental colitis (Elabd et al., 2018; Pan et al., 2022). In the present study, high doses of MOAE significantly increased the gene expression of mechanical barrier factors and chemical barrier factors and inhibited colonic inflammatory factors which may be related to the reduction in bacteria and toxic products that enter the immune barrier. Notably, MOAE may also increase the fecal water content of FC mice by decreasing the expression of colonic *Aqp3* and *Aqp4*, which in turn affects the entire intestinal microecosystem.

As an important environmental factor of the intestinal microecosystem, research on the role of the intestinal microbiota in constipation has received increasing attention. Intestinal microecological dysbiosis can lead to various functional gastrointestinal disorders, especially constipation. However, the mechanisms by which the gut microbiota and their metabolites affect intestinal motility are still poorly understood. In the present study, we found that MOAE can affect the gut microbial community structure and composition.

At the family level, Bacteroidaceae was the most noteworthy dominant microbial group, and MOAE reversed the significant increase of Bacteroidaceae in loperamide-induced FC mice, which is consistent with some clinical observations. For example, a higher relative abundance of Bacteroidacea was observed in constipated pediatric patients than in healthy children (Niu et al., 2020). Similarly, MOAE also reversed the significant increase in Clostridiaceae in FC mice induced by loperamide. Enrichment of Clostridiaceae in patients with slow transit constipation (Yu et al., 2023) and in loperamide-induced constipated rats (Huang et al., 2022) was also observed; Clostridiaceae bacteria are also considered to be characteristic of arthritis (Wang et al., 2017). Our correlation analysis also found that Bacteroidaceae and Clostridiaceae were significantly and negatively associated with the laxative phenotype. Although many studies support the hypothesis that Ruminococcaceae is a potentially beneficial organism, MOAE significantly reduces its abundance. A clinical study found that the relative abundance of Bacteroidaceae and Ruminococcaceae in chronic constipation patients with uncoordinated defecation was higher than that in chronic constipation patients with coordinated defecation (Yu et al., 2023), which laterally supports our findings. These results suggest that Bacteroidaceae, Clostridiaceae and Ruminococcaceae might play an important role in MOAE defecation at the family level, especially the dominant taxon Bacteroidaceae. Of course, we cannot exclude other taxa that are significantly altered by MOAE, and it is difficult for us to access their association with constipation.

Through a systematic literature review, we found that among the genus-level microbial groups significantly affected by MOAE, *Alloprevotella*, *Bacteroides*, *Butyricimonas*, *Muribaculum*, *Ruminococcus*, *Clostridium_sensu_stricto_1*, *Desulfovibrio* and *Butyricicoccus* were associated with constipation. Among them, *Alloprevotella*, *Bacteroides*, *Muribaculum* and *Butyricimonas* belong to the phylum Bacteroidetes. Many of these organisms are opportunistic pathogens. They were significantly enriched in loperamide-induced FC mice and significantly inhibited by MOAE.

*Bacteroides*, one of the most abundant members of the intestinal tract in most mammals, plays an important role in normal intestinal physiology and is most closely related to constipation. Oligosaccharides, konjac glucomannan, and yellow tea extracts all significantly reduced the abundance of *Bacteroides* in animals with constipation (Wang et al., 2017; Hayeeawaema et al., 2020; Cao et al., 2021). The results of clinical studies have also shown that FC patients have a higher abundance of *Bacteroides*, especially elderly FC patients (Guo et al., 2020; Wang et al., 2023). A study evaluating the safety and efficacy of fecal microbiota transplantation for chronic FC suggested that a high abundance of *Bacteroides* may be one of the causes of constipation (Tian et al., 2020). However, the genus *Bacteroides* is diverse and complex. Colonization with *B. thetaiotaomicron*, which was designed to produce tryptamine, led to accelerated gastrointestinal transport in GF mice (Bhattarai et al., 2018). *B. ovatus* can consume tryptophan and glutamate *in vitro* and synthesize the neuroactive compounds glutamine and GABA (Horvath et al., 2022).

Beneficial interactions with the host may be mediated by polysaccharide A or outer membrane vesicles of non-toxigenic *B. fragilis,* whereas systemic inflammation may be induced by toxigenic *B. fragilis* toxins or lipopolysaccharides (Sun et al., 2019); *B. fragilis*, on the other hand, requires only one step to turn from good to bad (Valguarnera and Wardenburg, 2020). Our correlation analysis revealed not only the correlation of *Bacteroides* with phenotypic indicators but also the significant correlation of *Bacteroides* with gastrointestinal motility factors, serum neurotransmitters, intestinal inflammatory factors and barrier factors, which further supports the hypothesis that a high abundance of *Bacteroides* causes functional constipation.

*Alloprevotella*, *Muribaculum,* and *Butyricimonas* have also been associated with constipation to varying degrees. It has been reported that elderly FC patients have a higher abundance of *Butyricimonas* (Guo et al., 2020), while *Muribaculum* is more abundant in complement 3 knockout mice with a constipation phenotype (Choi et al., 2021), and bamboo shavings-derived O-acetylated xylan alleviates constipation in mice while also reducing the levels of the potentially pathogenic bacteria *Alloprevotella* (Huang et al., 2022). Although the relative abundance of these microorganisms is low, their role cannot be ignored, and they also show a strong correlation with various types of host indicators.

*Ruminococcus* is a genus of Ruminococcaceae that is similar to *Bacteroides*. *Ruminococcus* was significantly enriched in loperamide-induced FC mice, while MOAE inhibited this genera. The relationship between microbes of the genus *Ruminococcus* and gastrointestinal motility is complex and seems to be paradoxical. Clinical studies have concluded that the relative abundance of *Ruminococcus* is higher in FC patients (Guo et al., 2020), and it is positively correlated with medical history in patients with slow-transit constipation (Tian et al., 2021); however, *R. gnavus* can play a pathogenic role in diarrhoeal irritable bowel syndrome by increasing 5-HT biosynthesis and promoting intestinal motility (Zhai et al., 2023). However, correlation analysis revealed not only the correlation between *Ruminococcus* and constipation phenotypic indicators but also the significant correlation between *Ruminococcus* and gastrointestinal motility factors, serum neurotransmitters, intestinal inflammatory factors, intestinal barrier factors and short-chain fatty acids, all of which support previous findings in clinical studies. Therefore, we speculate that the paradox may be related to the specificity of species function.

In the present study, another conditional pathogen, *Clostridium_sensu_stricto_1*, was significantly inhibited by MOAE. *Clostridium_sensu_stricto_1* was strongly associated with intestinal inflammation (Wang et al., 2018; Ma et al., 2022), which is also supported by our correlation analysis results. The significant correlations of *Clostridium_sensu_stricto_1* with the constipation phenotype, gastrointestinal hormones, neurotransmitters, intestinal motility factors, intestinal inflammatory factors and barrier factors in FC mice support its role in the relief of constipation by MOAE.

In addition to the significantly inhibited microbial taxa mentioned above, a number of microbial taxa were significantly enriched by MOAE treatment during the mitigation of FC, including *norank_f_Lachnospiraceae*, *Lachnospiraceae_UCG-010*, *Colidextribacter*, *Desulfovibrio*, *UCG-003*, *Family_XIII_UCG-001*, *Butyricicoccus*, *norank_f_norank_o_norank_c_Clostridia*, and *Tyzzerella*. Although we did not review their direct association with constipation, some of these taxa may play important roles in alleviating inflammation and protecting the intestinal barrier.

*Butyricicoccus* is a genus of butyrate-producing bacteria. The relative abundance of *Butyricicoccus* in the stool of patients with inflammatory bowel disease is low. Administration of *B. pullicaecorum* attenuated TNBS-induced colitis in rats, and the supernatant of *Butyricoccus* cultures enhanced intestinal epithelial barrier function (Eeckhaut et al., 2013). *Cannabis sativa* L. aqueous extracts significantly elevated the abundance of *B.* spp. while relieving constipation (Li R. et al., 2022; Li C. et al., 2022; Li L. et al., 2022). Our correlation analysis results also support the idea that *Butyricoccus* might play a beneficial role in the process of FC mitigation by MOAE.

To date, there are few reports on *Tyzzerella.* However, it has been reported that *Tyzzerella* can produce a large number of aromatic amines through the action of aromatic amino acid decarboxylase (Sugiyama et al., 2022), and many aromatic amines are able to promote intestinal motility. Our correlation analysis showed that *Tyzzerella* was positively correlated with GTR and various intestinal barrier factors and negatively correlated with FBST, SS and various gastrointestinal motility factors. Together, these results suggest that *Tyzzerella* might also play an important role in FC alleviation by MOAE.

Most studies have observed a possible facilitative role of *Desulfovibrio* in the development of constipation (Xu et al., 2022), and some drugs have been shown to reduce the abundance of *Desulfovibrio* in the treatment of constipation (Liu et al., 2019; Jiang et al., 2020). However, it has also been shown that *D. vulgaris* from the genus *Desulfovibrio* is an effective producer of acetic acid, which may be effective in reducing hepatic steatosis in mice by producing acetic acid and regulating hepatic lipid metabolism (Hong et al., 2021). In addition, some probiotics or prebiotics could also significantly enhance the abundance of *Desulfovibrio* in mice (Cui et al., 2022; Dong et al., 2022). Our results suggest that *Desulfovibrio* may play a beneficial role in the alleviation of FC by MOAE, as *Desulfovibrio* was significantly positively correlated with GTR and intestinal barrier factors and negatively correlated with FBST, gastrointestinal motility factors, and short-chain fatty acid content.

In addition, several other microbial taxa were significantly enriched under MOAE treatment, including *norank_f_Lachnospiraceae*, *Lachnospiraceae_UCG-010*, *Colidextribacter*, *UCG-003*, *Family_XIII_UCG-001*, and *norank_ f_norank_o_norank_c_Clostridia*. They also showed many correlations with host indicators, but due to the lack of literature, we cannot objectively comment on their role in the mitigation of FC by MOAE.

Microbes themselves affect gastrointestinal motility, and metabolites and components of microbes interact with the ENS. SCFAs can act directly on colonic and ileal smooth muscle (Rondeau et al., 2003), and SCFAs can also regulate 5-HT biosynthesis, thereby affecting colonic peristalsis (Fukui et al., 2018). Notably, the modulation of intestinal motility by SCFAs may be biphasic (Zhu et al., 2014), with low concentrations of SCFAs promoting intestinal motility and high concentrations of SCFAs triggering intestinal motility disorders (Shaidullov et al., 2021). In summary, the regulation of gut microbes and their metabolites is important for MOAE treatment of loperamide-induced constipation.

In summary, we explored the mechanism of MOAE in improving FC induced by loperamide from multiple perspectives related to the ENS and the intestinal microecosystem. We believe that MOAE could relieve constipation through regulating the ENS-ICCs-SMCs network, intestinal inflammation, the intestinal barrier, intestinal aquaporins, cecal SCFAs and the gut microbiota. Interestingly and importantly, MOAE could affect the entire intestinal microecosystem by inhibiting the growth of some key “constipation-causing” microbiota, such as Bacteroidaceae, Clostridiaceae, *Bacteroides*, and *Ruminococcus*, and promoting that of other important “constipation-curing” microbiota, such as *Butyricoccus*, *Tyzzerella*, and *Desulfovibrio*. Meanwhile, the correlations established between the important different gut microbes and core host parameters provide a basis for further elucidating the gut microbiota-host metabolism relationship in FC.

## Data availability statement

The raw reads of 16S rRNA gene sequence data were deposited into the NCBI Sequence Read Archive (SRA) database under BioProject accession number PRJNA994313.

## Ethics statement

The animal study was approved by Animal Ethics Committee of Yunnan Agriculture University. The study was conducted in accordance with the local legislation and institutional requirements.

## Author contributions

XG: Conceptualization, Data curation, Formal analysis, Funding acquisition, Methodology, Visualization, Writing – original draft, Writing – review & editing. WQY: Data curation, Formal analysis, Methodology, Writing – original draft. SiL: Methodology, Writing – original draft. ShL: Writing – original draft. WXY: Methodology, Writing – original draft. SS: Methodology, Writing – original draft. JS: Conceptualization, Funding acquisition, Supervision, Writing – review & editing. YZ: Conceptualization, Supervision, Visualization, Writing – review & editing. YT: Conceptualization, Supervision, Visualization, Writing – review & editing.
