# Supplementary material for: Moringa oleifera leaf alleviates functional constipation via regulating the gut microbiota and the enteric nervous system in mice
Source: Front Microbiol. 2023 Dec 20;14:1315402. doi: 10.3389/fmicb.2023.1315402 (PMC10773919; doi:10.3389/fmicb.2023.1315402)
Supplement: Supplementary file 1 [file Data_Sheet_1.docx]

**Supplementary materials**

**Title: *Moringa oleifera* leaf alleviates functional constipation via the microbiota - enteric nervous system axis in mice**

Xiaoyu Gao^1,2,3#^, Weiqian Yang^2,4#^, Sijin Li^5^, Shuangfeng Liu^2,3^, Weixing Yang^2^, Shuang Song^2^, Jun Sheng^3*^, Yan Zhao^6*^, Yang Tian^1, 3*^

1. Yunnan Key Laboratory of Precision Nutrition and Personalized Food Manufacturing, Yunnan Agricultural University, Kunming 650201, China

2. College of Food Science and Technology, Yunnan Agricultural University, Kunming 650201, China

3. Engineering Research Center of Development and Utilization of Food and Drug Homologous Resources, Ministry of Education, Yunnan Agricultural University, Kunming 650201, China

4. Hotel Management Department, Chongqing Vocational Insititute of Tourism, Chongqing 409000, China

5. College of Pu’er Tea, West Yunnan University of Applied Sciences , Puer 665099, China

6. Division of Science and Technology, Yunnan Agricultural University, Kunming 650201, China

# contributed equally to this work.

* Address correspondence to Jun Sheng, shengj@ynau.edu.cn; Yan Zhao, 2021013@ynau.edu.cn(+86-15908792807); or Yang Tian, tianyang@ynau.edu.cn.

Supplementary Materials list:

● Table S1 The main nutritional components of Moringa Oleifera leaf aqueous extract (MOAE)

● Table S2 Primers sequences used for quantitative PCR analysis of gene expression

● Table S3 Classification of phytochemical composition of MOAE

● Table S4 Phytochemical composition of MOAE

● Fig. S1 Effects of MOAE on loperamide-induced constipation symptoms in mice

● Fig. S2 Effects of MOAE on the mRNA expression of intestinal inflammation, intestinal barrier, and intestinal antimicrobial peptide in ileum

● Fig. S3 Effect of MOAE on rarefaction crurves and alpha diversity of the gut microbiota in FC mice

● Fig. S4 Effect of MOAE on the cecum microbial composition in FC mice.

● Fig. S5 Linear discriminant analysis

● Fig. S6 Heat maps showing correlations between the specific gut bacteria (at the family level and genus level) and core host parameters.

**Table S1. The main nutritional components of *Moringa Oleifera* leaf aqueous extract (MOAE)**

| Composition | Content | Method | Reference |
| --- | --- | --- | --- |
| Moisture | 6.92% | Direct drying | [1] |
| Ash | 16.80% | Weigh after burning | [2] |
| Fat | 1.75% | Acid-hydrolysis | [3] |
| Protein | 20.20% | Kjeldahl | [4] |
| Carbohydrate | 54.33% | Calculated | [5] |
| Crude Polysaccharide | 5.14% | Spectrophotometry | [6] |
| Total acid | 0.40% | pH potentiometric titration | [7] |
| Phosphorus | 0.79% | Inductively coupled plasma optical emission spectrometer | [8] |
| Potassium | 5.11% |  |  |
| Calcium | 4.18% |  |  |
| Magnesium | 1.55% |  |  |
| Manganese | 284.00 mg/kg |  |  |
| Zinc | 32.00 mg/kg |  |  |
| Iron | 77.60 mg/kg |  |  |
| Copper | 2.92 mg/kg |  |  |
| Sodium | 316.00 mg/kg |  |  |
| Vitamin C | 720.00 mg/kg | HPLC | [9] |
| Phytochemical composition | - | HPLC-QQQ-MS/MS | [10] |

**Reference:**

[1] GB 5009.3-2016, National food safety standard-Determination of moisture in food[S]. (In Chinese)

[2] GB 5009.4-2016, National food safety standard-Determination of ash in food[S]. (In Chinese)

[3] GB 5009.6-2016, National food safety standard-Determination of fat in food[S]. (In Chinese)

[4] GB 5009.5-2016, National food safety standard-Dedetermination of protein in food[S]. (In Chinese)

[5] GB/Z 21922-2008, Basic terms of food nutrients[S]. (In Chinese)

[6] NY/T 1676-2008, Determination of crude polysaccharide in edible fungi[S]. (In Chinese)

[7] GB 12456-2021, National food safety standard-Determination of total acids in foods[S]. (In Chinese)

[8] GB 5009.268-2016, National Food Safety Standard-Determination of Multiple Elements in Food [S]. (In Chinese)

[9] GB 5009.86-2016. National food safety standard-Determination of ascorbic acid in food [S]. (In Chinese)

[10] Doppler, M., Kluger, B., Bueschl, C., Schneider, C., Krska, R., Delcambre, S., Hiller, K., Lemmens, M., & Schuhmacher, R. (2016). Stable Isotope-Assisted Evaluation of Different Extraction Solvents for Untargeted Metabolomics of Plants. International journal of molecular sciences, 17(7), 1017. https://doi.org/10.3390/ijms17071017.

**Phytochemical composition determination by widely targeted metabolomics**

A total of 10 mg freeze-dried MOAE powder was combined in a centrifuge tube with 2 small steel balls and 500 μL extract (methanol: water, volume ratio 1: 2). The mixture was precooled at 40 ℃, including the internal standard. The mixture was mixed for 30 s, homogenized at 35 Hz for 4 min, and placed in an ice water bath for ultrasonic homogenization for 5 min. The ultrasonic homogenization was repeated 3 times. Then the mixture was incubated overnight at 4 ℃ on the homogenizer. Next, the mixture was centrifuged for 15 min at 12,000 rpm (centrifugal force 13,800 g, radius 8.6 cm) at 4 ℃. The supernatant was carefully filtered through a 0.22 μm microporous membrane. One hundred microliters of each sample was mixed into QC samples and stored at -80 ℃ until computer analysis.

The target compositions were separated by EXIONLCSystem (SCIEX) performance liquid chromatography (EXIONLCSystem) and a Waters UPLC liquid chromatography column. The liquid chromatography phase was an aqueous solution containing 0.1% formic acid, and phase B was acetonitrile. The temperature of the column incubator was 40 °C, the temperature of the automatic injector was 4 °C, and the injection volume was 2 μL. A Sciex QTrap 6500+ (Sciex Technologies) was used for assay development. Typical ion source parameters were as follows: ion spray voltage: +5500/-4500 V, curtain gas: 35 psi, temperature: 400 ℃, ion source gas 1:60 psi, ion source gas 2: 60 psi, DP: ± 100 V.

SCIEX Analyst Work Station Software (Version 1.6.3) was used for MRM data acquisition and processing. MS raw data (wiff) files were converted to the TXT format using MS conventer. An in-house R program and database were used for peak detection and annotation.

**Table S2. Primers sequences used for quantitative PCR analysis of gene expression**

| Gene name | Primer | Sequence (5' to 3') |
| --- | --- | --- |
| *Occludin* | Forward | ATGTCCGGCCGATGCTCTC |
|  | Reverse | TTTGGCTGCTCTTGGGTCTGTAT |
| *ZO-1* | Forward | TTTTTGACAGGGGGAGTGG |
|  | Reverse | TGCTGCAGAGGTCAAAGTTCAAG |
| *Muc-2* | Forward | ACGTGTCATATTTGCACCTCT |
|  | Reverse | TCAACATTGAGAGTGCCAACT |
| *TNF-α* | Forward | TATGGTCAAGGTCTTCTCGGGTCG |
|  | Reverse | AGTGCTGTCATGCGTTGGAAGTCTC |
| *MCP-1* | Forward | AGCCATCCGACATTCTTC |
|  | Reverse | GCCTATGCCTTCCACTTT |
| *IL-1β* | Forward | AAGAAGGTGGTGAAGCAGG |
|  | Reverse | GAAGGTGGAAGAGTGGGAGT |
| *IL-6* | Forward | GTTCTCTGGGAAATCGTGGA |
|  | Reverse | TGTACTCCAGGTAGCTA |
| *Defa* | Forward | GGTGATCATCAGACCCCAGCATCAGT |
|  | Reverse | AAGAGACTAAAACTGAGGAGCAGC |
| *Lyz1* | Forward | GCCAAGGTCTACAATCGTTGTGAGTTG |
|  | Reverse | CAGTCAGCCAGCTTGACACCACG |
| *Pla2g2* | Forward | AGGATTCCCCCAAGGATGCCAC |
|  | Reverse | CAGCCGTTTCTGACAGGAGTTCTGG |
| *Reg3g* | Forward | TTCCTGTCCTCCATGATCAA |
|  | Reverse | CATCCACCTCTGTTGGGTTC |
| *smMLCK* | Forward | AGAAGTCAAGGAGGTAAAGAATGATGT |
|  | Reverse | CGGGTCGCTTTTCATTGC |
| *c-Kit* | Forward | CAGAAACCCATGTATGAAGT |
|  | Reverse | CTTTCCAAAACTCAGCCTGT |
| *SCF* | Forward | CTCAGTTTTGTGGCTTCGTTTA |
|  | Reverse | CTACCATGTCCGATACTACGAC |

**Table S3. Classification of phytochemical composition of MOAE**

| **Classification of compounds** | **Relative abundance**  **(%)** |
| --- | --- |
| Flavonoids | 30.14 |
| Amino Acids | 22.23 |
| Nucleotides | 13.47 |
| Lipids | 13.17 |
| Alkaloid | 5.99 |
| Phenols | 3.01 |
| Vitamins and Organic acids | 1.71 |
| Phenylpropanol | 0.35 |
| Tryptamine | 0.33 |
| Hydroxy acids | 0.21 |

**Table S4. Phytochemical composition of MOAE**

| CAS | FORMULA | NAME | Relative content(%) |
| --- | --- | --- | --- |
| 63-91-2 | C9H11NO2 | L-Phenylalanine | 11.84% |
| 482-35-9 | C21H20O12 | Isoquercitrin | 8.73% |
| 480-10-4 | C21H20O11 | Astragalin | 6.38% |
| 5536-17-4 | C10H13N5O4 | Vidarabine | 5.33% |
| 7665-99-8 | C10H12N5O7P | Guanosine 3',5'-cyclic monophosphate | 4.56% |
| 74-79-3 | C6H14N4O2 | L-Arginine | 2.59% |
| 634-01-5 | C10H12N5O6P | Adenosine 2',3'-cyclic phosphate | 2.57% |
| 147-85-3 | C5H9NO2 | Proline | 2.55% |
| 1818-71-9 | C10H13N5O5 | Crotonoside | 1.70% |
| 3681-93-4 | C21H20O10 | Vitexin | 1.55% |
| 501-97-3 | C9H10O3 | Phloretic acid | 1.27% |
| 120-72-9 | C8H7N | Indole | 1.22% |
| 134-20-3 | C8H9NO2 | Methyl anthranilate | 1.11% |
| 52-52-8 | C6H11NO2 | Pipecolic acid | 1.08% |
| 104-87-0 | C8H8O | 4-Methylbenzaldehyde | 1.01% |
| 14091-11-3 | C9H10ClNO2 | 2-Chloro-DL-Phenylalanine | 0.96% |
| 535-83-1 | C7H7NO2 | Trigonelline | 0.89% |
| 2623-91-8 | C4H9NO2 | D-alpha-Aminobutyric acid | 0.86% |
| 153-18-4 | C27H30O16 | Rutin | 0.74% |
| 60-12-8 | C8H10O | Phenethyl alcohol | 0.69% |
| 14639-25-9 | C6H5NO2 | 2-Picolinic acid | 0.67% |
| 28338-59-2 | C27H30O15 | Cyanidin 3-rutinoside | 0.64% |
| 480-43-3 | C16H14O5 | Isosakuranetin | 0.61% |

**
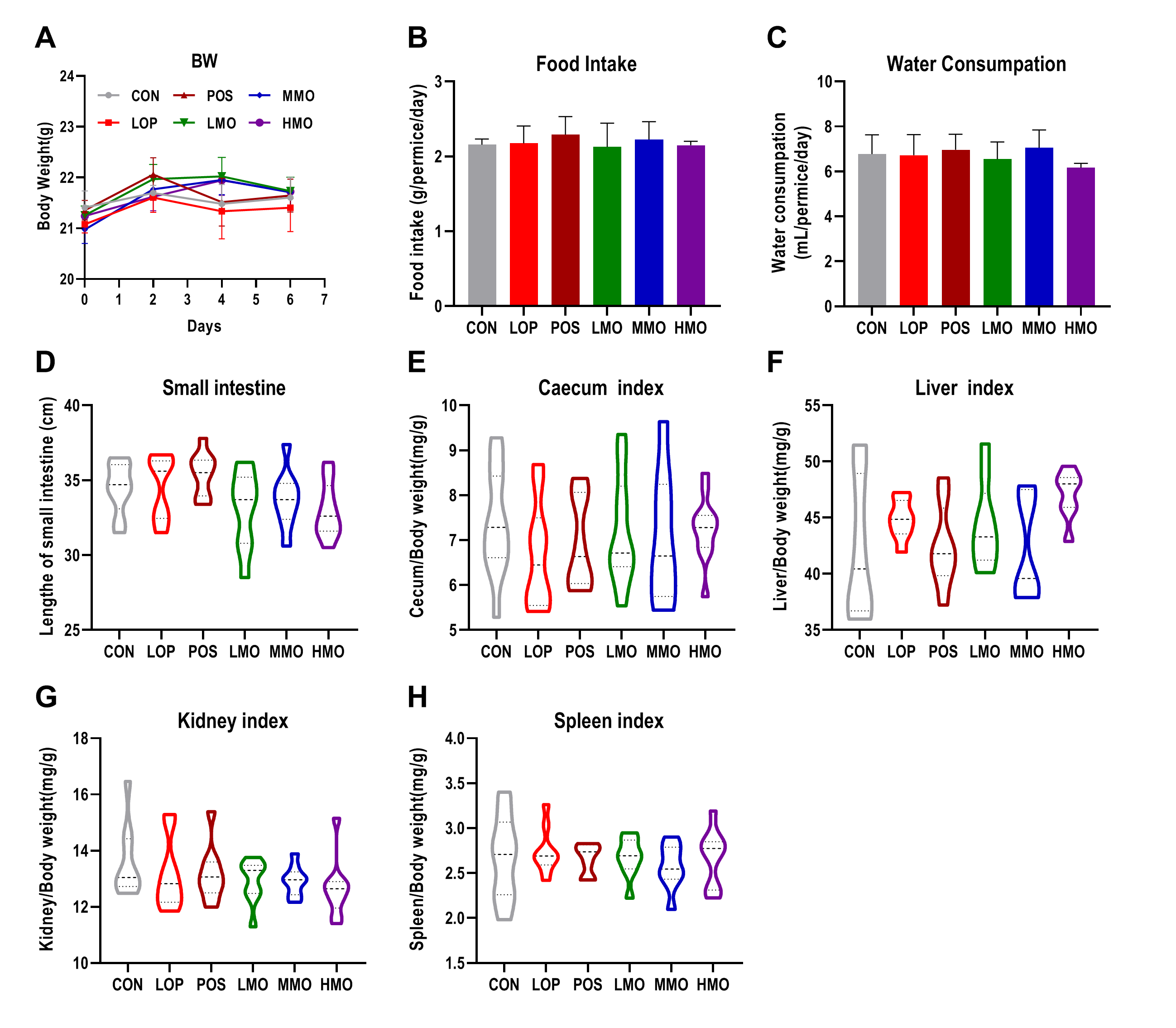
**

**Fig. S1. Effects of MOAE on loperamide-induced constipation symptoms in mice.** (A) The body weight change; (B) Food intake; (C) Water consumption; (D) The length of small intestine; (E) Cecum index; (F) Liver index; (G) Kidney index, (H) Spleen index

**
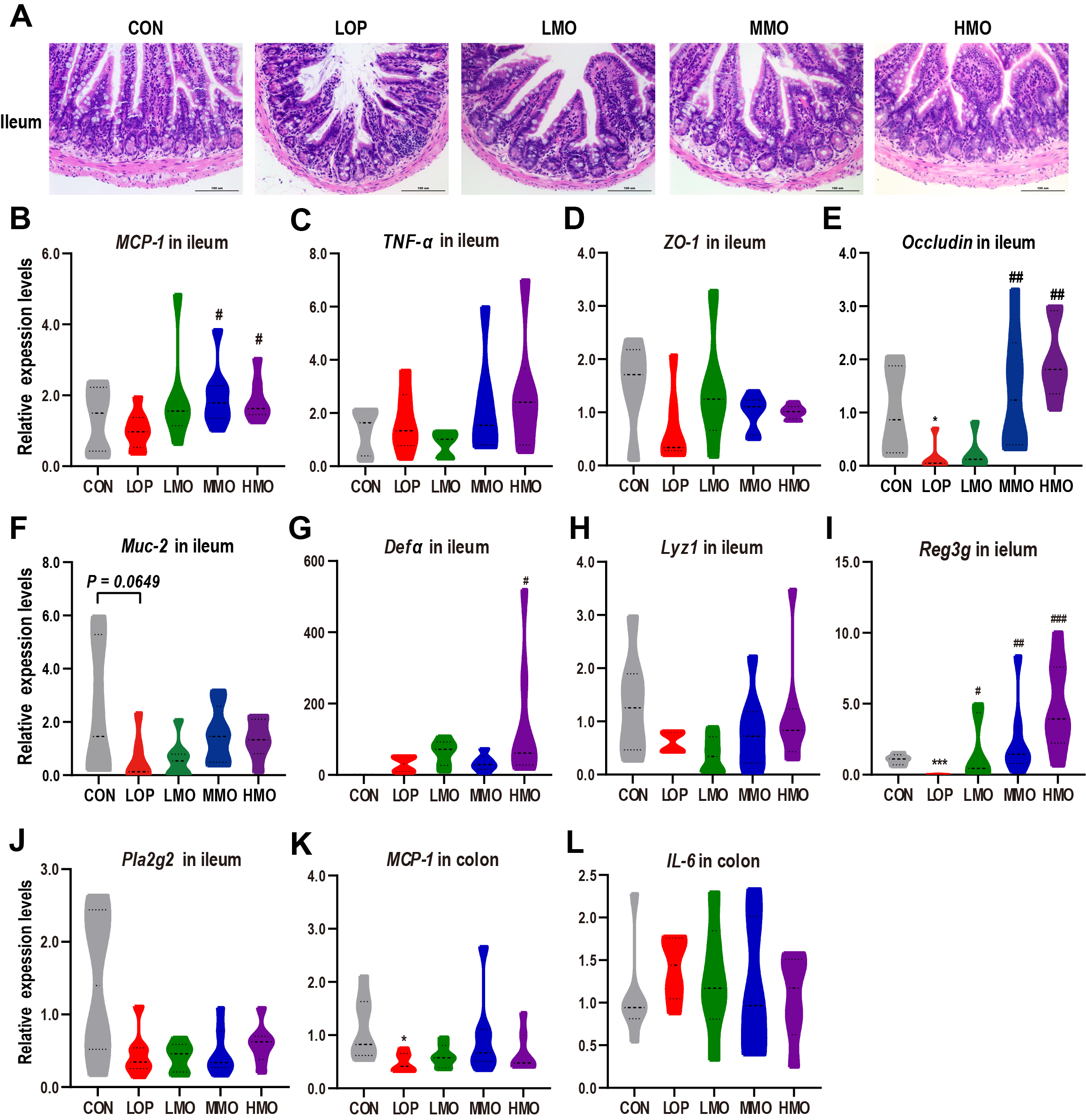
**

**Fig. S2 Effects of MOAE on the mRNA expression of intestinal inflammation, intestinal barrier, and intestinal antimicrobial peptide in ileum**

(A) Photomicrographs of H&E-stained distal ileum sections.

(B-C) Expression of inflammation genes in the ileum, including *MCP-1* and *TNF-α*.

(D-F) Expression of intestinal barrier genes in the ileum, including *ZO-1*, *Occludin*, and *Muc2*.

(G-J) Expression of AMP genes in the ileum, including *Defa*, *Lyz1*, *Reg3g*, and *Pla2g2*.

(K-L) Expression of *MCP-1* and *IL-6* in the colon.

The data are expressed as the means ± SEMs (*n* = 8). *, compared with the CON group; #, compared with the LOP group. *, *P* < 0.05; ***, *P* < 0.001. #, *P* < 0.05; ##, *P* < 0.01; ###, *P* < 0.001.

**
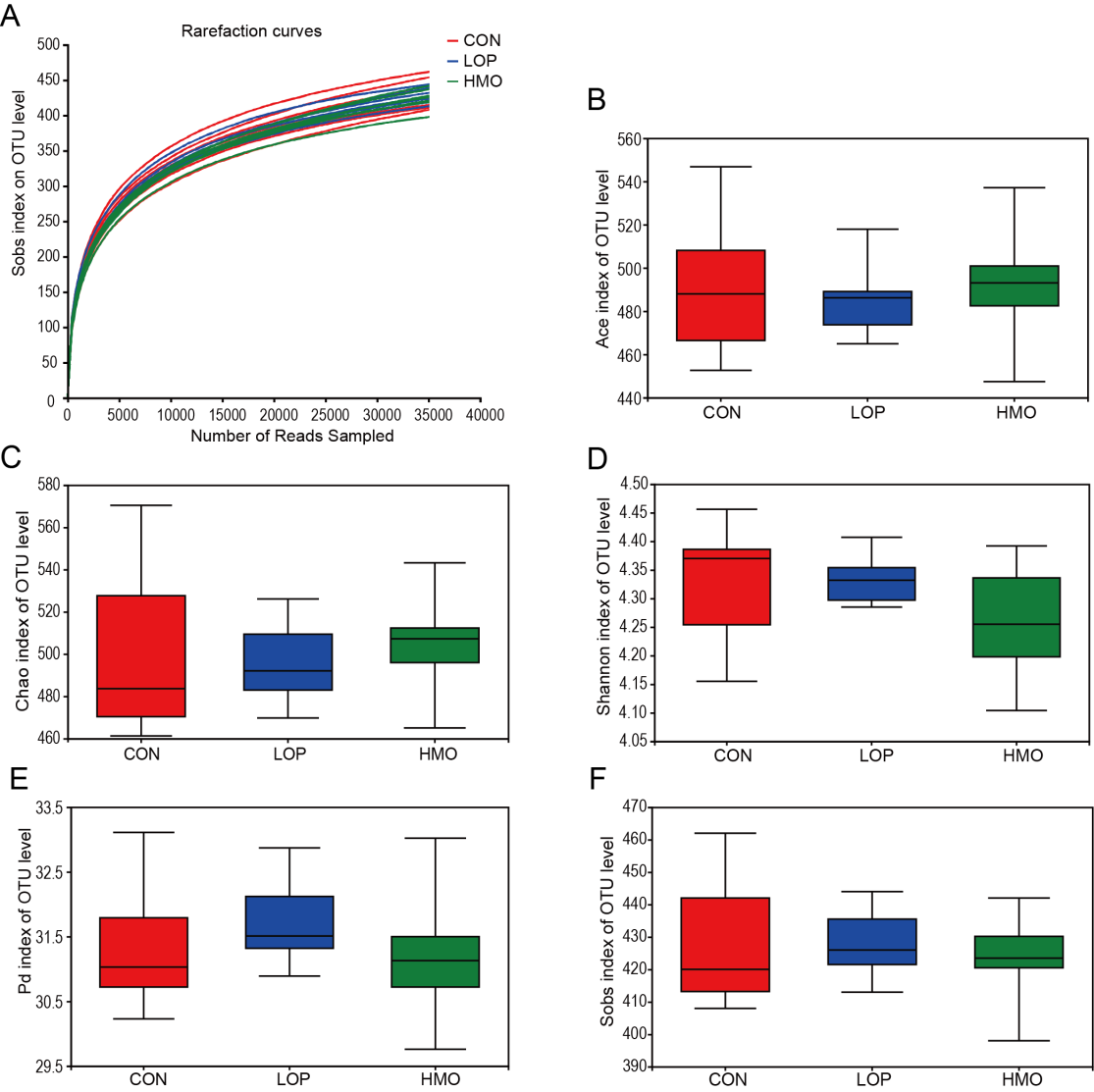
**

**Fig. S3 Effect of MOAE on rarefaction curves and alpha diversity of the gut microbiota in FC mice.**

**
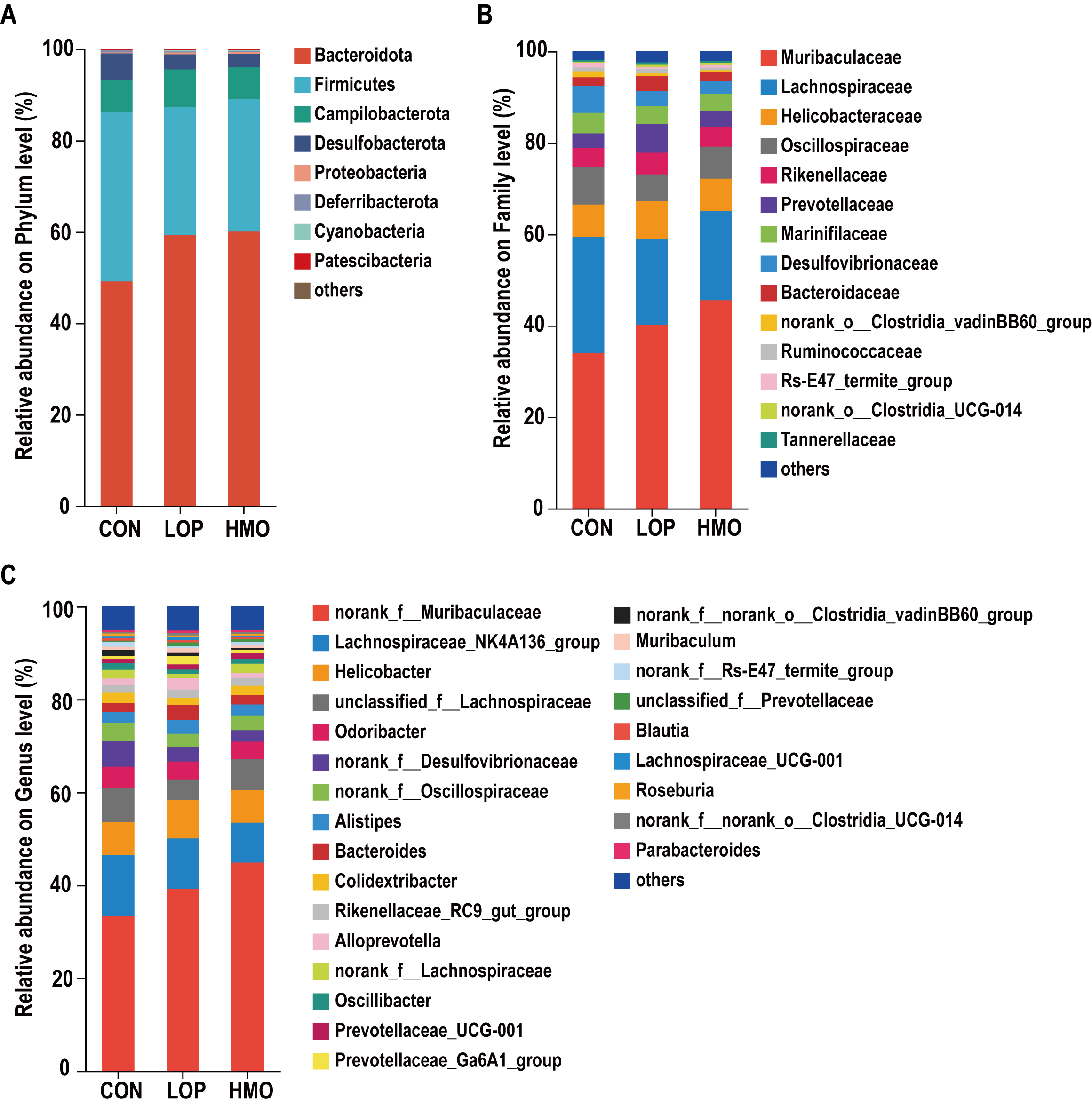
**

**Fig. S4 Effect of MOAE on the cecum microbial composition in FC mice. (A) Phylum level. (B) Family level. (C) Genus level.**

**
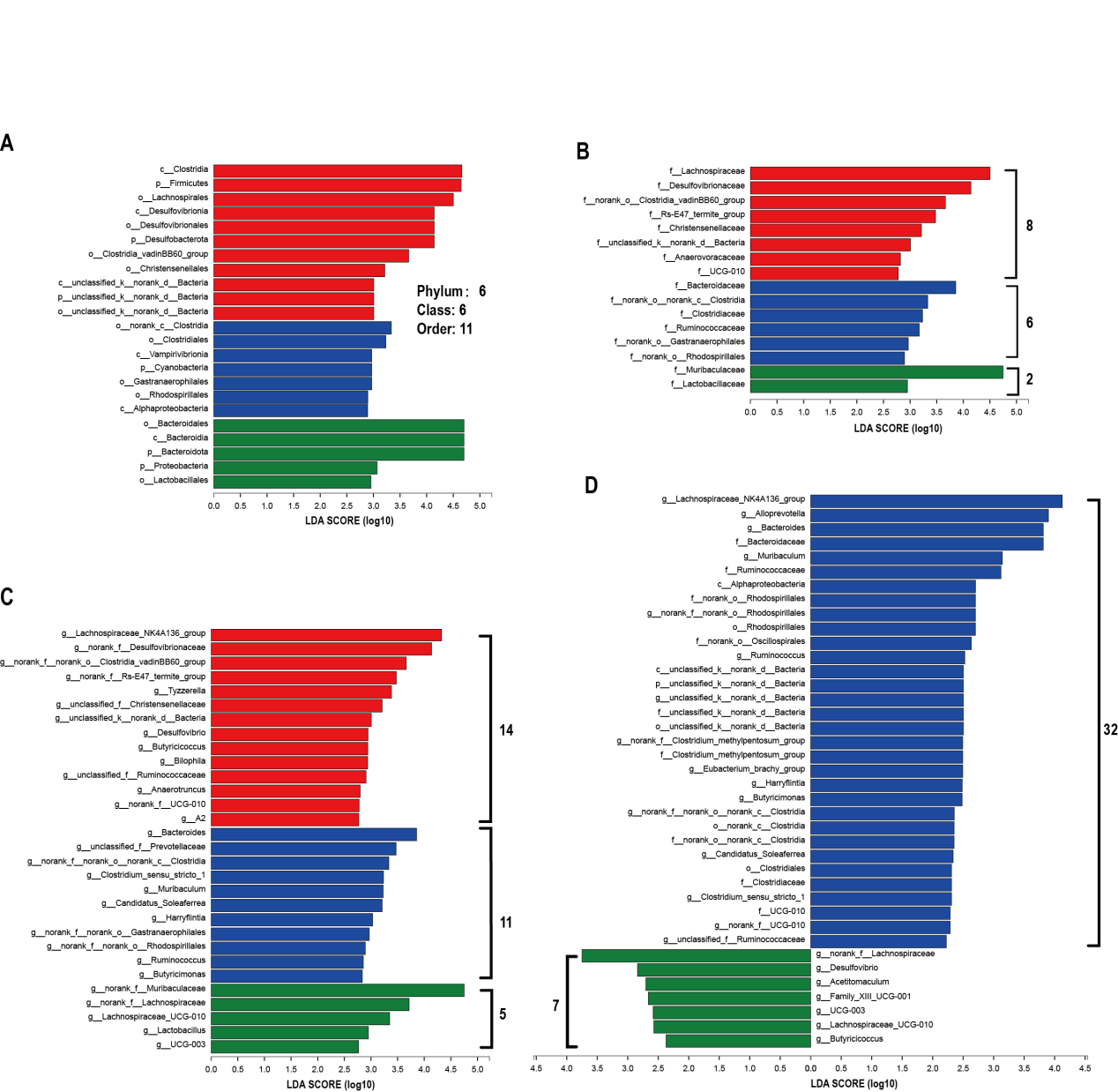
**

**Fig. S5 Linear discriminant analysis (LDA score ≥ 2.0).**

(A-C) LDA based on the CON, LOP and HMO groups, at the phylum, class and order level(A), at the family level (B), at the genus level(C). (D) LDA based on the LOP and HMO groups.

**
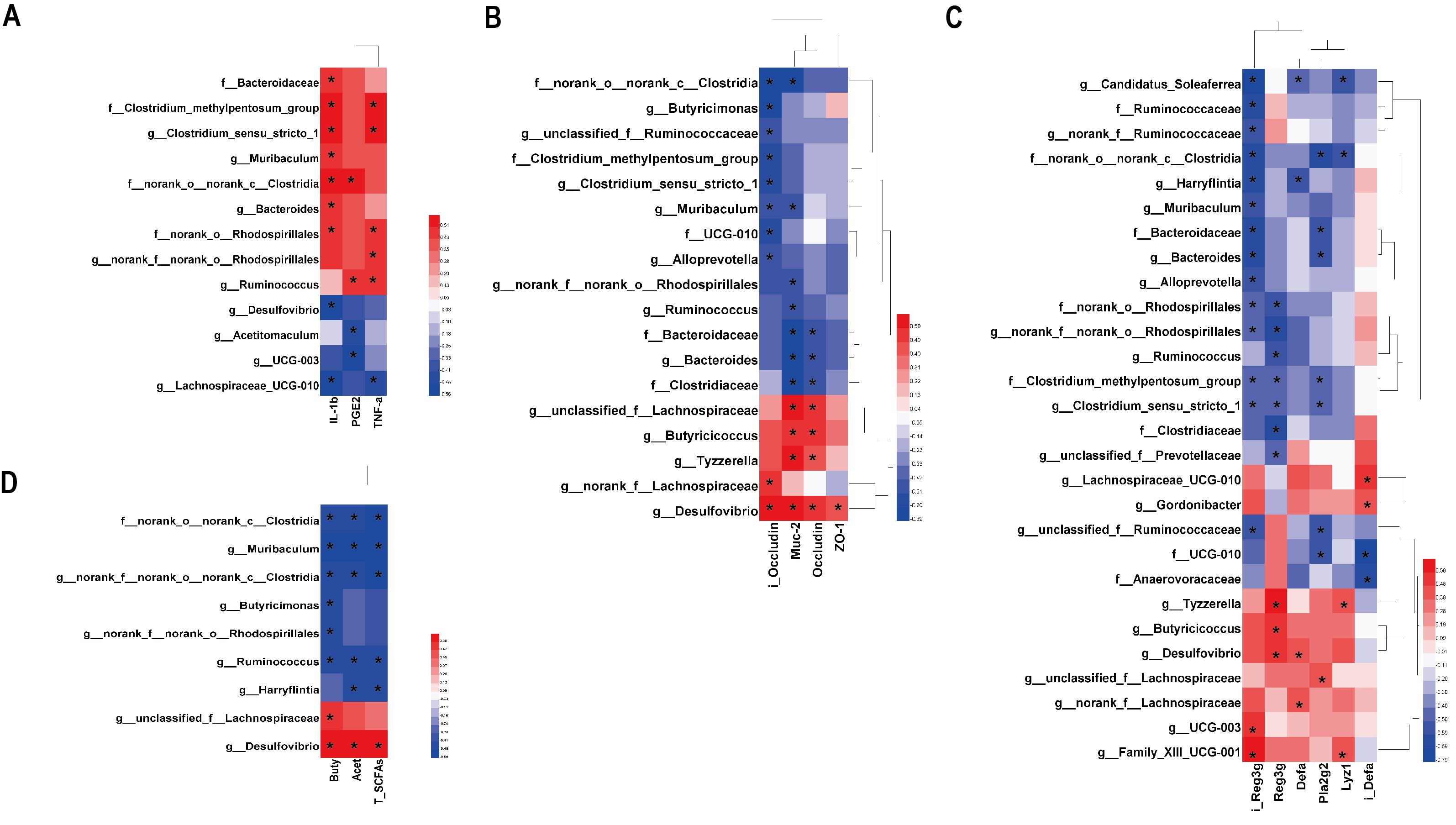
**

**Fig. S6** Heat maps showing correlations between the specific gut bacteria (at the family level and genus level) and core host parameters.

(A) Correlation between gut bacteria and intestinal inflammation factors, including the mRNA expression of *TNF-α*, *IL-1β* and *PGE2* in the colon. (B) Correlation between gut bacteria and gut barrier-related indicators, including the mRNA expression of *Occludin*, *Muc2*, *ZO-1* in the colon and *Occludin* in the ileum (*iOccludin*), respectively. (C) Correlation between gut bacteria and intestinal AMPs; *iDefa* and *iReg3g* indicate the mRNA expression of *Defa* and *Reg3g* in the ileum, *Defa*, *Pla2g2*, *Reg3g* and *Lyz1* indicate their mRNA expression in the colon. (D) Correlation between gut bacteria and SCFAs in cecal contents, including total SCFAs, acetic acid, and butyric acid. The color at each intersection indicates the value of the *r* coefficient; *P*-values were adjusted for multiple testing according to the *Bonferroni* and *Hochberg* procedures. * indicates a significant correlation between these two parameters (*P <* 0.05).
